# Overcoming barriers: Modelling the effect of potential future changes of organized breast cancer screening in Italy

**DOI:** 10.1177/09691413231153568

**Published:** 2023-02-10

**Authors:** Nadine Zielonke, Carlo Senore, Antonio Ponti, Marcell Csanadi, Harry J de Koning, Eveline A M Heijnsdijk, Nicolien T van Ravesteyn

**Affiliations:** 1 Department of Public Health, Erasmus MC, University Medical Center Rotterdam, Rotterdam, The Netherlands; 2 Epidemiology and screening Unit – CPO, University Hospital Città della Salute e della Scienza, Turin, Italy; 3 Syreon Research Institute, Budapest, Hungary

**Keywords:** Breast cancer screening, microsimulation, Italy, adherence, screening interval, cost-effectiveness

## Abstract

**Objectives:**

Organized breast cancer screening may not achieve its full potential due to organizational and cultural barriers. In Italy, two identified barriers were low attendance in Southern Italy and, in Italy as a whole, underscreening and overscreening in parts of the eligible population. The objective of this study was to identify potential changes to overcome these barriers and to quantify their costs and effects.

**Methods:**

To assess the impact of potential measures to improve breast cancer screening in Italy, we performed an evaluation of costs and effects for increasing adherence for Southern Italy and harmonizing screening intervals (biennial screening) for the whole of Italy, using an online tool (EU-TOPIA evaluation tool) based on the MIcrosimulation SCreening ANalysis (MISCAN) model.

**Results:**

Increasing adherence in Southern Italy through investing in mobile screening units has an acceptable cost-effectiveness ratio of €9531 per quality-adjusted life year gained. Harmonizing the screening interval by investing in measures to reduce opportunistic screening and simultaneously investing in mobile screening units to reduce underscreening is predicted to gain 1% fewer life-years, while saving 19% of total screening costs compared to the current situation.

**Conclusions:**

Increasing adherence in Southern Italy and harmonizing the screening interval could result in substantial improvements at acceptable costs, or in the same benefits at lower costs. This example illustrates a systematic approach that can be easily applied to other European countries, as the online tools can be used by stakeholders to quantify effects and costs of a broad range of specific barriers, and ways to overcome them.

## Introduction

Early detection and diagnosis of breast cancer are effective ways to reduce mortality of the disease.^1–3^ In Italy, organized breast cancer screening began in the late 1990s in Florence, with the invitation of 50–69-year-old women. More regions followed and current Italian guidelines recommend inviting women aged 50–69 to undergo mammography every two years, in accordance with EU Recommendations.^4^ There is no nationally organized breast cancer screening program in Italy, but regional health authorities have a mandate to implement breast cancer screening programs. All resident women in the target age range must be invited, and all screening tests, ascertainment and treatments are free of charge.^5,6^ The performance of all regional screening programs is monitored by the national centre for screening monitoring (Osservatorio Nazionale Screening, ONS).

Owing to several factors (barriers), cancer screening programs may fail to achieve their full potential. Within the European Union funded EU-TOPIA^7^ (Towards improved screening for breast, cervical and colorectal cancer in all of Europe) project, a self-assessment tool (Supplemental Methods Part 1) was developed to identify barriers to the optimal operation of population-based cancer screening programs.^8^ For Italy, barriers have been identified (Supplemental Table A1) that are common to those identified in other countries.^9^ The introduction of screening programs in Italy has been slow and characterized by profound geographical differences.^10,11^ Despite efforts to reduce and overcome heterogeneity in screening between regions, Southern Italy is showing low attendance rates that do not reach the European Commission recommended minimum of 70%.^5,10,12,13^ In 2018, the invitation coverage was only 59% and the adherence to the invitation was 38%.^14^ Looking at Italy nationally, opportunistic and organized screening for breast cancer coexist, which complicates the attainment of comprehensive information about screening coverage.^10,15^ Opportunistic screening might also lead to a screening interval that is not in line with EU Recommendations,^4^ because some eligible women are using opportunistic screening options in addition to the program, thus leading to overscreening and inefficient utilization of resources. At the same time, other eligible women have extended screening intervals due to a lack of human and financial resources, thus leading to underscreening.

The objective of this paper is to identify potential future changes that can be initiated in breast cancer screening programs – as well as their costs and effects – to overcome identified barriers (i.e. low attendance in Southern Italy, underscreening and overscreening) and to improve screening performance in Italy. In this modelling study, we quantify the harms, benefits and costs of changes to organized screening strategies for breast cancer in Italy.

## Methods

### MISCAN breast model

We used the MIcrosimulation SCreening ANalysis (MISCAN) model, which has been described in detail elsewhere.^16^ In brief, MISCAN simulates individual life histories and assesses consequences of introducing a screening program on these life histories using the Monte Carlo method. Possible events are birth and death, onset of a pre-clinical ductal carcinoma in situ (DCIS), transitions between disease states, participation in screening, and screen or clinical detection of a cancer.^17,18^ Originally, the model was calibrated to the Dutch situation.^18^

We adjusted and calibrated MISCAN to reflect the Italian demography (i.e. age distribution of the population and life expectancy), breast cancer incidence, screening adherence (invitation and attendance) and performance (detection rate and stage distribution), and potential differences in the natural history of breast cancer. In developing the Italian model, we used a specific calibration process described previously.^16^ Data on breast cancer incidence and stage distribution were provided as pooled data from 38 regional cancer registries (AIRTUM: Associazione Italiana Registri Tumori; Italian Association of Cancer Registries), while data about attendance and outcomes of screening programs were provided by ONS and the Italian group for mammography screening (GISMa). We calibrated the model by fitting the onset rate by age and the stage-specific dwell times against the observed incidence rate for the last available period, 2006–2009. The extent of opportunistic screening was included based on expert opinion.^19^ We first validated the model replicating data that were used in the calibration process (internal validation). Then, we externally validated the model against best evidence based on a recently published systematic review on breast cancer mortality reductions due to screening.^2^

### EU-TOPIA evaluation tool

Effects of chosen measures to further improve breast cancer screening in Italy were assessed using the EU-TOPIA evaluation tool, which is based on MISCAN. The tool is an online platform that allows stakeholders to use country-specific data to quantify future harms and benefits of different cancer screening scenarios in their country.^20^ Stakeholders can download an Excel template to provide country-specific data on population demography, breast cancer epidemiology, the screening program and treatment of breast cancer. After uploading the data, they can simulate the current cancer screening programs and impacts of potential changes in screening protocols (e.g. changing target ages or screening attendance). A detailed user's guide can be found in Supplemental Methods Part 2.

The evaluation tool was developed for three cancer sites (breast, cervical and colorectal cancer). Four country-specific benchmark models represent four European regions: the Netherlands (Western Europe), Slovenia (Eastern Europe), Finland (Northern Europe), and Italy (Southern Europe). In this paper, we use the breast cancer version for Southern Europe (Italy). Registered users can find detailed information about the cancer-specific versions on the tool's website.^21^

### Simulated screening scenarios

We evaluated the effect of two barriers on screening outcomes and how to potentially overcome them: first, we focused on low screening rates in Southern Italy. Second, we focused on the whole of Italy, characterized by sub-populations screened at different intervals due to opportunistic screening and capacity barriers (Table 1). Screening performance indicators were derived from data provided in the EU-TOPIA monitoring tool.

**Table 1.** Overview of assumptions of screening behaviour and simulated screening scenarios, per barrier and scenario.

Barrier 1: Southern Italy					
	Scenario 1	Scenario 2	Scenario 3	Scenario 4	Scenario 5
	Current screening	+20%	+30%	+40%	+100%
Screening interval (years)	2.0	2.0	2.0	2.0	2.0
Adherence by age^a^					
50–54	11.5%	31.5%	41.5%	51.5%	100.0%
55–59	14.0%	34.0%	44.0%	54.0%	100.0%
60–64	15.2%	35.2%	45.2%	55.2%	100.0%
65–69	14.8%	34.8%	44.8%	54.8%	100.0%
*Average*	*13.9%*	*33.9%*	*43.9%*	*53.9%*	*100.0%*
Barrier 2: Italy (national)					
	Sub-group 1: overscreened^b^		Sub-group 2: underscreened^b^		Sub-group 3: biennial^b^
	Scenario 1^c^	Scenario 2	Scenario 1^c^	Scenario 2	Scenario 1^c^
Screening interval (years)	1.0	2.0	2.4	2.0	2.0
Adherence by age^a^					
50–54	100.0%	100.0%	100.0%	100.0%	100.0%
55–59	100.0%	100.0%	100.0%	100.0%	100.0%
60–64	100.0%	100.0%	100.0%	100.0%	100.0%
65–69	100.0%	100.0%	100.0%	100.0%	100.0%

^a^ Adherence is specified as the proportion (%) of the target population screened in the index year after invitation.

^b^ The proportions of the sub-groups of the female population eligible for screening are 20% for sub-group 1, 30% for sub-group 2 and 50% for sub-group 3.

^c^ For each barrier/sub-group, scenario 1 represents the current screening scenario.

First, we looked at the particular situation in Southern Italy, which comprises Abruzzo, Molise, Campania, Basilicata, Puglia, Calabria, Sicilia and Sardegna. We simulated the current screening (scenario 1) assuming biennial mammography screening from 50 to 69 years with an adherence in 2013 (report year) of 14%. Subsequently, we investigated the impact of investing in mobile screening units. Previous work indicates that short distances to facilities and increased screening capacities could increase adherence to screening.^22^ Thus, we simulated four additional scenarios with increased adherence by 20%, 30%, 40% and 100% – equally added to the reported adherence of each 5-year age-group.

For the second barrier, we split the female Italian population into sub-groups. We assumed that of all eligible women (ages 50–69), 20% are screened annually due to a combination of organized screening and opportunistic screening utilization. Thirty percent of all eligible women are assumed to be screened with an extended screening interval of 28 months, while half (50%) of all eligible women are screened biennially. We simulated the sub-groups separately and calculated weighted averages of outcomes to obtain national results.

For each sub-group, we simulated two scenarios: current screening with existing barrier (scenario 1) and biennial screening from 2020 onwards without this barrier (scenario 2). For the overscreened sub-group, we investigated the impact of investing in legislative measures, as well as in a training and information campaign for radiologists and general practitioners (GPs), to lower opportunistic screening. For the underscreened sub-group, we investigated the impact of investing in mobile screening units across the whole country to increase screening capacity and to subsequently decrease the screening interval.

### Model outcomes and cost-effectiveness analyses

Using the EU-TOPIA evaluation tool, life histories of 10 million women were simulated. For each simulated scenario, we estimated breast cancer outcomes in (Southern) Italy for women aged 40–100 years in the period 2020–2050. Outcomes include the predicted number of breast cancer cases (DCIS and invasive), breast cancer deaths, breast cancer mortality reduction (% compared to no screening), number of false positives and overdiagnoses (as % of screen-detected breast cancers), resources required by the breast cancer screening program (number of screening tests and referrals), number of screens needed to prevent one breast cancer death, and number of false positives per breast cancer death prevented.

Subsequently, we performed a cost-effectiveness analysis for each simulated scenario from a healthcare payer perspective and calculated direct medical costs including costs of screening, diagnostics and treatment. For costs of a screening mammogram, diagnosis and treatment by cancer stage we used previously reported estimates.^23–25^ In order to increase screening adherence in Southern Italy (i.e. to overcome barrier 1), we considered an investment of 10 mobile screening units (at €180,000 each). In order to overcome barrier 2, we considered two different investments separately: investing €2.5 million to stop opportunistic screening and investing €18 million for 100 mobile screening units. An overview of all costs and disutilities is shown in Table 2.

**Table 2.** Costs and disutilities used in the cost-effectiveness analysis, per barrier and scenario.

Variable	Costs (€)							Disutilities	Duration
	Barrier 1		Barrier 2						
	Scenario 1	Scenario 5	Sub-group 1		Sub-group 2		Sub-group 3		
Investment costs^a^	−	1,800,000^b^	−	2,500,000^c^	−	18,000,000^d^	−		
Screening invitation letter	3	3	1.5^e^	3	3	3	3		
Mammography	45	45	60^f^	45	45	45	45	0.006	1 week
Diagnosis	190	190	252^f^	190	190	190	190	0.105	1 month
Treatment of DCIS	9204	9204	9204	9204	9204	9204	9204	0.100	2 years
Treatment of T1A	11,816	11,816	11,816	11,816	11,816	11,816	11,816	0.100	2 years
Treatment of T1B	14,483	14,483	14,483	14,483	14,483	14,483	14,483	0.100	2 years
Treatment of T1C	14,272	14,272	14,272	14,272	14,272	14,272	14,272	0.250	2 years
Treatment of T2+	20,522	20,522	20,522	20,522	20,522	20,522	20,522	0.250	2 years
Death from breast cancer								0.400	6 months

^a^ Investment costs are specified as an additional investment secondary to the resources routinely spent for the operation of the current program, in order to overcome the respective barrier.

^b^ Investment to finance 10 mobile screening units at €180,000 each.

^c^ Investment to finance legislative measures, training and information campaign for radiologists and GPs.

^d^ Investment to finance 100 mobile screening units at €180,000 each.

^e^ We assume half of the exams in this group to be performed outside the organized setting. Those women will not receive an invitation, thus these costs are lower for this sub-group.

^f^ We adapted the costs of the screening process (mammography and diagnosis) for the sub-group of overscreened women based on results from Mantellini and Lippi.^24^ They detected cost differences between opportunistic and organized screening in seven screening centres in Italy. Based on their findings, we assumed that the exams in this group that are performed outside the organized setting (50%) are 1.65 times more expensive.

For each scenario, costs and quality-adjusted life-years (QALYs) gained were calculated. Costs and effects were discounted at 3% per year. Subsequently, in order to compare screening scenarios, cost-effectiveness ratios (CERs) were calculated for strategies that try to overcome each barrier (scenario 5 for barrier 1, scenario 2 for barrier 2), thus screening 100% of the eligible women in Southern Italy and screening all eligible women every two years. CERs are the difference in costs divided by the difference in QALYs between a strategy and the current situation (Scenario 1 for each barrier). The CER of a strategy therefore reflects costs required to generate one additional QALY, compared to the current strategy.

### Sensitivity analysis

We performed three sensitivity analyses. First, we varied the number of mobile screening units invested in for barrier 1 (Southern Italy). For barrier 2, we used observed or estimated adherence of each sub-group instead of 100%: 75% adherence in the overscreened group, 45%–50% adherence in the underscreened group, and the observed 55%–60% national average for biennial screening. In addition, we varied the sub-group distribution for the simulations of barrier 2 by assuming higher proportions of the population being underscreened or overscreened.

## Results

Screening women in Southern Italy biennially from age 50 to 69 with an adherence to screening of 14% (current screening), would lead to 556,000 breast cancer diagnoses (DCIS and invasive) for women aged 40–100 years in the period 2020–2050, of which 7% would be screen-detected (Table 3). We estimated a mortality reduction of 4.6% compared to no screening, with a total of 7184 breast cancer deaths prevented and 6200 life-years gained, accompanied by 3.8% overdiagnosis and 336,000 false-positive results. Fully overcoming the barrier of low adherence, that is, increasing adherence to 100% (scenario 5), is predicted to increase the share of screen-detected cancers to 31%, mortality reduction to 17.4% and would prevent 15,736 additional breast cancer deaths, while the percentage of overdiagnosis would remain the same (3.8%).

**Table 3.** Summary of screening outcomes and cost-effectiveness results for barrier 1 (low adherence in Southern Italy), by simulated scenario.

Screening outcomes^a^					
	Scenario 1	Scenario 2	Scenario 3	Scenario 4	Scenario 5
Number of screen tests (×1000)	5714	+14,263	+12,338	+16,506	+35,495
Number of referrals (×1000)	373	+539	+808	+1077	+2316
Number of false positives (×1000)	336	+495	+745	+997	+2172
Number of BC diagnoses^b^ (% of screen detected)	556 (7%)	+6 (14%)	+9 (18%)	+11 (21%)	+18 (31%)
Number of BC deaths (×1000)	149	−5	−7	−9	−16
BC mortality reduction (%) compared to no screening	4.6	+2.9	+4.2	+5.4	+10.1
Prevented BC death	7184	+4491	+6517	+8371	+15,736
Overdiagnosis (%)^c^	3.8	−0.1	+/−	+0.1	-+/−
Cost-effectiveness results^d^					
	Scenario 1	Scenario 2	Scenario 3	Scenario 4	Scenario 5
Primary screening costs	19,557,465	+19,042,995	+28,553,655	+38,052,315	+81,823,335
Costs for diagnostic follow-up	13,371,310	+4,611,182	+6,950,391	+9,317,104	+20,438,783
Costs for cancer care	539,714,842	−1,820,303	−2,685,599	−3,549,885	−7,659,337
Net costs compared to no screening	15,838,465	+21,887,873	+32,890,447	+43,909,534	+94,773,681
Life-years gained vs no screening	6200	+3000	+4300	+5600	+10,600
QALY gained vs no screening	6277	+2868	+4106	+5345	+10,072
CER^e^	-	7633	8010	8216	9410

BC: breast cancer; QALY: quality-adjusted life-year; CER: cost-effectiveness ratio; +/−: approximately the same results as scenario 1.

^a^ Results represent the difference in screening outcomes or costs between each scenario (2 to 5) and current screening (scenario 1) and are reported for women aged 40–100 in 2020–2050.

^b^ Total of clinically and screen-detected DCIS and invasive breast cancers (percentage of screen detected in brackets).

^c^ Overdiagnosis is defined as women that would not have been diagnosed during their lives if they had never been screened as a percentage of all screen-detected women.

^d^ The results are presented for 1 million women followed from 2020 over their life-times. Both costs and effects are discounted with 3% per year.

^e^ CERs were computed as ratio between incremental net costs and benefits (QALY) compared to the current scenario (scenario 1).

Investing in 10 mobile screening units increased the total screening costs (for primary screening, diagnostic follow-up and cancer care) from €573 million to €667 million (+17%, Table 3). Compared to the current screening strategy, the CER was €9410 per QALY gained for adherence of 100%.

 Table 4 presents the results for barrier 2 for the whole of Italy. Continuing current screening – characterized by a combination of screening intervals (i.e. weighted average of three sub-groups) – would lead to 1.3 million breast cancer diagnoses (DCIS and invasive) for women aged 40–100 years in 2020–2050, of which 32% would be screen detected. Compared to a no-screening situation, breast cancer mortality reduction would be 24.6%, a total of 94,998 breast cancer deaths would be prevented and 27,560 life-years would be gained (Table 4, weighted average scenario 1). If described actions were to be initiated to achieve biennial screening of all eligible women from 2020 onwards (weighted average of scenario 2 of each sub-group), a similar number of breast cancer diagnoses and share of screen-detected cancers is predicted. Moreover, overcoming this barrier would lead to an unchanged breast cancer mortality reduction and percentage of overdiagnosis (5%) while false-positive results would decrease by 13%. Nearly the same number of breast cancer deaths would occur and 310 fewer life-years would be gained between 2020 and 2050 compared to current screening. Screening all eligible women in Italy every two years is predicted to lead to 11% more screen tests and less expensive cancer care, and therefore to a saving of €122 million in total screening costs. While overcoming this barrier leads to a substantial decrease in referrals (−12%), false positive results (−13%) and costs (−18%), almost all benefits would be maintained. Therefore, maintaining a mix of screening intervals leads to a CER of €162,799 per QALY, compared to a situation where all eligible women are screened biennially. Thus, remaining in the current screening scenario is very cost-inefficient.

**Table 4.** Summary of screening outcomes and cost-effectiveness results for barrier 2 (screening intervals), by simulated scenario.

Screening outcomes^a^		
	Weighted average (scenario 1)	Weighted average (scenario 2)
Number of screen tests (×1000)	114,589	+13,153
Number of referrals (×1000)	8131	−980
Number of false positives (×1000)	7714	−971
Number of BC diagnoses^b^ (% of screen detected)	1294 (32%)	+2 (32%)
Number of BC deaths (×1000)	291	+2
BC mortality reduction (%) compared to no screening	24.6	+/−
Prevented BC death	94,998	−1149
Overdiagnosis^c^	5.0	+/−
Cost-effectiveness results^d^		
	Weighted average^d^ (scenario 1)	Weighted average^d^ (scenario 2)
Primary screening costs	129,107,349	−25,599,189
Costs for diagnostic follow-up	34,990,611	−6,376,965
Costs for cancer care	480,691,757	−89,562,403
Net costs compared to no screening	127,936,053	−30,440,225
Life-years gained vs no screening	27,560	−310
QALY gained vs no screening	27,402	−187
CER^e^	162,799	3578

BC: breast cancer; QALY: quality adjusted life-year; CER: cost-effectiveness ratio; +/−: approximately the same results as scenario 1.

^a^ Results represent the difference in screening outcomes or costs between scenario 2 and current screening (scenario 1) and are reported for women aged 40–100 in 2020–2050.

^b^ Total of clinically and screen-detected DCIS and invasive breast cancers (percentage of screen detected in brackets).

^c^ Overdiagnosis is defined as women that would not have been diagnosed during their lives if they had never been screened as a percentage of all screen-detected women.

^d^ The results are presented for 1 million women followed from 2020 over their life-times. Both costs and effects are discounted with 3% per year.

^e^ CERs were computed as ratio between incremental net costs and benefits (QALY) compared to the current scenario (scenario 1).

### Sensitivity analysis

Varying investment costs by varying the number of mobile screening units (investing in 100 or 1000 units instead of 10) hardly changed the total screening costs or CERs (Table 5). Even an investment of €180 million would still be cost-efficient in order to overcome barrier 1. Model-predicted base-case screening outcomes (weighted averages) were not particularly sensitive to assuming complete adherence for barrier 2. Harmonizing the screening interval throughout Italy would remain cost-saving (i.e. maintaining a mix of screening intervals would cost €83,072 per QALY gained) even when observed adherence per sub-group would be applied. Assuming sub-groups would account for 30% (overscreened), 20% (underscreened) and 50% (biennial) led to savings of €41,569 per QALY gained compared to a situation where barrier 2 is present. Assuming sub-groups would account for 20% (overscreened), 50% (underscreened) and 30% (biennial) led to savings of €31,586 per QALY gained compared to a situation where barrier 2 is present.

**Table 5.** Sensitivity analysis by simulated barrier and adapted parameter.

Screening outcomes									
	Barrier 1^a^		Barrier 2^b^						
			Current screening (scenario 1)			Overcoming the barrier (scenario 2)			
	100 units	1000 units	Observed adherence	Sub-groups^c^ 30/20/50	Sub-groups^c^ 20/50/30	Observed adherence	Sub-groups^c^ 30/20/50	Sub-groups^c^ 20/50/30	
Number of screen tests	+/−	+/−	−20%	+40%	+22%	−40%	+/−	−5%	
Number of referrals	+/−	+/−	−37%	+10%	−3%	−40%	+/−	−5%	
Number of false positives	+/−	+/−	−38%	+11%	−4%	−41%	+/−	−5%	
Number of BC diagnoses^d^ (% of screen detected)	+/−	+/−	+/− (32%)	+/− (32%)	+/−	+/−	−1%	−2%	
Number of BC deaths	+/−	+/−	+9%	−1%	+1%	+9%	−1%	+1%	
BC mortality reduction (%) compared to no screening	+/−	+/−	−26%	+4%	−2%	−29%	+2%	−4%	
Prevented BC death	+/−	+/−	−26%	+4%	−2%	−29%	+2%	−4%	
Overdiagnosis^e^	+/−	+/−	+8%	+3%	−1%	+8%	+1%	+2%	
Cost-effectiveness results									
	Barrier 1^a^		Barrier 2^b^						
			Current screening (scenario 1)			Overcoming the barrier (scenario 2)			
	100 units	1000 units	Observed adherence	Sub-groups^c^ 30/20/50	Sub-groups^c^ 20/50/30	Observed adherence	Sub-groups^c^ 30/20/50		Sub-groups^c^ 20/50/30
Primary screening costs	+/−	+/−	−34%	+14%	+/−	−37%	−5%		−5%
Costs for diagnostic follow-up	+/−	+/−	−28%	+12	+/−	−30%	−4%		−4%
Costs for cancer care	+/−	+/−	−17%	−19%	+/−	+2%	+/−		+/−
Net costs compared to no screening	+1%	+16%	−36%	+17%	+/−	−41%	−3%		−6%
Life-years gained vs no screening	+/−	+/−	−28%	+4%	+/−	−28%	−3%		−3%
QALY gained vs no screening	+/−	+/−	−27%	+4%	+/−	−28%	−3%		−3%
CER^f^	€9572	€11,180	€83,072	€41,569	€31,586				

BC: breast cancer; QALY: quality adjusted life-year; CER: cost-effectiveness ratio; +/−: approximately the same results as base-case analysis.

^a^ Results are presented as percentage difference between base-case scenario 5 (100% adherence) and varied investment costs based on number of mobile screening units.

^b^ Results are presented as percentage difference in the weighted average between the respective sensitivity analysis and the base-case analysis.

^c^ The proportions of the sub-groups of the female population eligible for screening that are overscreened/underscreened/screened biennially.

^d^ Total of clinically and screen-detected DCIS and invasive breast cancers (percentage of screen detected in brackets).

^e^ Overdiagnosis is defined as women that would not have been diagnosed during their lives if they had never been screened as a percentage of all screen-detected women.

^f^ CERs were computed as ratio between incremental net costs and benefits (QALY) compared to the current scenario (scenario 1).

## Discussion

We investigated two major barriers to breast cancer screening programs in (Southern) Italy and ways to overcome them by initiating feasible changes. We found that investing in overcoming these barriers would lead to better long-term outcomes. The employed online tools could be effective and reliable resources for European policymakers aiming for informed policy-making on cancer screening in their country.

In Italy, as in most European countries, opportunistic and organized screening coexist. The extent of opportunistic screening in Italy is estimated to be 19%.^19^ This is of concern, since organized screening programs are more likely to be attended by socio-economically disadvantaged women,^3,6,26,27^ low coverage in organized screening programs is associated with health and social inequalities,^27^ and opportunistic screening is associated with higher screening costs.^24^ Moreover, it has been shown that coverage of (organized) screening is of key importance in order to reach full public health potential.^28–30^ A recent study conducted in Italy showed that reducing social inequalities associated with organized breast screening programs is not only related to increasing early diagnoses but also to improving access to effective treatments.^26^ Therefore, organized screening programs may have the potential to eliminate at least some barriers encountered by disadvantaged women and reduce inequities.^31,32^ Assuming that mobile mammography units help to increase participation,^33–36^ we showed that this strategy would be cost-effective in regions with low screening adherence. Moreover, earlier studies^33^ showed that decreasing geographical barriers is also an effective measure for decreasing social inequalities in participation in breast cancer screening for the total population.

To fully overcome barrier 1, increasing screening adherence among eligible Southern Italian women from 14% to 100% might seem like an unrealistic, unfeasible and probably undesirable attempt (i.e. a screening adherence of 100% probably conflicts with informed choice). However, we wanted to reflect the maximum effect of increasing adherence with our simulations. Instead of aiming at fully overcoming the barrier, our results show that even an adherence of 54% (+40%, scenario 4) would lead to a doubling of screening benefits.

A strength of our analysis is the presented systematic approach and stepwise process that can be easily followed or adapted by other European countries or stakeholders. The EU-TOPIA evaluation tool helps to estimate harms and benefits of the current screening program, and supports users to define and evaluate alternative screening scenarios reflecting on how key barriers can be overcome. Subsequently, modelling quantifies the long-term impact of proposed changes on screening outcomes. Continuous monitoring of screening activity would then permit to assess whether implemented changes actually achieved the expected impact. In addition, an important strength is that a calibrated and validated model and its transparent methods were used. Furthermore, the barriers analyzed in this study are common to different countries, as presented in two prior publications.^8,9^ Another strength of this paper is that it illustrates a pathway for evaluating long-term impact of these barriers on screening outcomes and also shows the effect of overcoming them by initiating practical solutions. Previous publications have shown similar examples for colorectal screening and cervical screening, highlighting that it is worth spending effort to remove barriers that affect the interval at which women participate in screening.^37,38^

There are three noteworthy limitations of our analysis. First, it is based on the barrier assessment of one group of key informants. Future analyses should also include views of local providers, in particular in countries where screening is decentralized. Second, there are more potential measures to limit the use of opportunistic screening or to increase screening adherence than the ones described in this paper. A recently published paper^6^ looked at very low attendance for breast and cervical cancer screening and the role of related determinants in the Southern Italian region, Campania. Their findings underline the urgent need for improved population education on cancer prevention and specifically on benefits and harms of cancer screening. Third, while our analysis focuses on the target age of 50–69 years, some regions in Italy also invite younger or older^14^ women to screening. However, we assume the effect of such regional disparities on our reported national results to be marginal.

## Conclusions

Our analysis shows that removing the most important barriers to the current Italian breast cancer screening programs could result in substantial improvements at acceptable costs. The used online tools are valuable for stakeholders to quantify benefits, harms and costs of early cancer detection in Europe. This Italian example illustrates a systematic approach and stepwise process that can be easily followed or adapted by other European countries or stakeholders.

## Supplemental Material

sj-docx-1-msc-10.1177_09691413231153568 - Supplemental material for Overcoming barriers: Modelling the effect of potential future changes of organized breast cancer screening in Italy Click here for additional data file. Supplemental material, sj-docx-1-msc-10.1177_09691413231153568 for Overcoming barriers: Modelling the effect of potential future changes of organized breast cancer screening in Italy by Nadine Zielonke, Carlo Senore, Antonio Ponti, Marcell Csanadi, Harry J de Koning, Eveline A M Heijnsdijk and Nicolien T van Ravesteyn in Journal of Medical Screening

sj-docx-2-msc-10.1177_09691413231153568 - Supplemental material for Overcoming barriers: Modelling the effect of potential future changes of organized breast cancer screening in Italy Click here for additional data file. Supplemental material, sj-docx-2-msc-10.1177_09691413231153568 for Overcoming barriers: Modelling the effect of potential future changes of organized breast cancer screening in Italy by Nadine Zielonke, Carlo Senore, Antonio Ponti, Marcell Csanadi, Harry J de Koning, Eveline A M Heijnsdijk and Nicolien T van Ravesteyn in Journal of Medical Screening
