# Supplementary material for: Overcoming barriers: Modelling the effect of potential future changes of organized breast cancer screening in Italy
Source: J Med Screen. 2023 Feb 10;30(3):134–41. doi: 10.1177/09691413231153568 (PMC10399099; doi:10.1177/09691413231153568)
Supplement: sj-docx-1-msc-10.1177_09691413231153568 - Supplemental material for Overcoming barriers: Modelling the effect of potential future changes of organized breast cancer screening in Italy [file sj-docx-1-msc-10.1177_09691413231153568.docx]

**Supplementary Methods**

**EU-TOPIA Barrier to effective screening tool (BEST)**

The European Union funded EU-TOPIA (Towards improved screening for breast, cervical and colorectal cancer in all of Europe) project aims to improve health outcomes and equity of breast, cervical and colorectal cancer screening programmes in ways that take full account of the different demographical, medical, political, economic and cultural contexts across Europe. The project aims to do so by providing national, regional, and local policymakers with tools to evaluate and quantify their cancer screening programmes. One of the tools created was a self-assessment process for the purpose of identifying the barriers to the optimal operation of population-based breast cancer screening programmes.
The online tool was used with workshop participants prior to and during an EU-TOPIA workshop in Turin, April 2019. This Excel version has been made available so that screening organisers, researchers and policy-makers in Europe can continue to use and apply the tool in future projects.

## Background to the tool

The tool is based on a health systems approach to evaluating health services. This means that all parts of the screening process are important from start to finish. We identified six sub-systems in our model: generation of knowledge and effectiveness, identification of the population at risk, maximising informed participation, successful operation of the programme, adequate follow-up and ensuring effective treatment for those that need it^1 2^.

## Aim of the tool

The aim of the tool is to enable screening organisers, researchers and policy-makers to make a self-assessment of their organised breast, cervical and colorectal cancer screening programmes to identify the most important barriers to effectiveness and equity. The tool also allows users to prioritise the most important barriers, also considering feasibility, and identify ways to overcome barriers.

## How to apply the tool

The tool should be applied separately for each cancer screening programme (breast, cervical and colorectal). The tool is the same for each cancer type but the barriers may differ between programmes. The tool has been developed primarily for use in countries, regions or municipalities where organised, population-based cancer screening programmes are already in place, or at least where a pilot programme is in operation. Where a programme has yet to be introduced, we suggest respondents consider the main barrier(s) to the introduction of such a programme and use this as the basis for discussions on how to overcome barriers.

Ideally, the tool should incorporate the views of all stakeholders, either by each stakeholder completing the tool separately (and the results amalgamated to determine an average) or by organising a stakeholder meeting to complete the tool using a consensus approach. The process of completing the tool (the thought processes involved and discussions) are as important as the results of the tool. Hence, it would be useful to make notes whilst completing the tool to capture any new insights raised from the discussion. Moreover, it should be emphasised that the barriers are the opinion of the respondents and that the impact of barriers varies, depending in part on measures taken to mitigate their effects.

## Key sections of the tool

Firstly, we would like you to rate the importance of each barrier that exists in your country in terms of its impact on programme effectiveness and equity. Secondly, we would like you to select the three most important barriers to overcome. Thirdly, we would like you provide any examples you have from your country of ways that barriers have been overcome (or at least reduced). The latter section was primarily of interest to the EU-TOPIA workshop for sharing knowledge across countries. However, this exercise may still be useful for discussions about how to overcome remaining barriers.

## What next?

The results of this self-assessment exercise will enable you to prioritise barriers to be reduced or overcome and whether the resource currently being allocated to overcoming barriers is being used effectively. In addition, this tool is part of several work packages from the EU-TOPIA project. Following on from this work package is another work package on 'road maps' to further develop and improve cancer screening programmes. The results from this activity inform the road maps. Please refer to the EU-TOPIA website for further information on the workshops and other tools available.

**Table A1: Results of the barrier assessment from an Italian stakeholder***

| **Sub-system** | **Barrier** | **Effect^1^** | **Equity^1^** | **Rank^3^** |
| --- | --- | --- | --- | --- |
| Knowledge | Issues with establishing protocols, processes and legal frameworks (e.g. inadequate national governance structure, professionals with relevant knowledge) | 3 | 3 |  |
|  | Screening guidelines and protocols are not regularly updated or updates are delayed (e.g. by complex administration procedures) | 2 | 1 |  |
| Identification | Population register is not accurate (e.g. not updated with changes of address) | 2 | 2 |  |
|  | Population register is not complete (e.g. some eligible people not included) | 2 | 2 |  |
| Participation | Some people have beliefs and values that lead to non-participation in screening programme | 4 | 4 | **1** |
|  | Some people experience practical issues that lead to non-participation in screening programme (e.g. inconvenient appointments, inadequate health insurance) | 3 | 4 |  |
|  | Inadequate public promotion of screening programme (e.g. primary care physicians are not sharing information or promoting screening) | 3 | 3 |  |
|  | Inadequate system for monitoring levels and patterns of screening participation (e.g. inequalities among some subgroups) | 3 | 2 |  |
|  | Inadequate response to low levels of uptake (informed participation) and patterns of screening participation (e.g. inequalities among some subgroups) | 4 | 4 |  |
| Operation | Inadequate information technology (IT) systems (e.g. disjointed systems) | 4 | 1 |  |
|  | Insufficient human, physical and/or financial resources to operate screening programme (e.g. limited capacity, organisational or logistical issues) | 4 | 3 | **2** |
|  | Inadequate adherence by providers to screening guidelines and protocols (e.g. opportunistic screening occurs outside the organised screening programme) | 4 | 4 | **3** |
|  | Inadequate system for monitoring operational aspects of the screening programme (e.g. quality of screening experiences of those who participate) | 3 | 2 |  |
|  | Inadequate response to address quality issues relating to the operation of the screening programme | 3 | 1 |  |
| Follow up | Insufficient human, physical and/or financial resources to conduct follow-up investigations for those that need it | 4 | 3 |  |
|  | Inadequate adherence by providers to follow-up guidelines and protocols (e.g. clinician's attitudes and established pattern of practice) | 4 | 1 |  |
|  | Inadequate system for monitoring people who require follow-up investigations but do not participate (e.g. due to personal beliefs or practical issues) | 2 | 1 |  |
|  | Inadequate response to people who require follow-up investigations but do not participate (e.g. due to personal beliefs or practical issues) | 2 | 1 |  |
|  | Inadequate sharing of follow-up information between national/regional screening organisations, providers of follow-up investigations and primary care | 2 | 1 |  |
| Treatment | Some people have beliefs and values that lead to them declining cancer treatment | 2 | 4 |  |
|  | Insufficient human, physical and/or financial resources to provide treatment to those that need it | 3 | 4 |  |
|  | Inadequate system for monitoring treatment information (e.g. treatment data not systematically linked to cancer screening data) | 4 | 2 |  |
|  | Inadequate sharing of treatment information between national or regional screening organisations, providers of cancer treatment and primary care | 4 | 2 |  |

*What is your job role? - Regional screening organisation representative, researcher

Does your country have an organised, population-based screening programme for breast cancer in operation? - Yes - regionally organised

Do you have expert knowledge of breast cancer screening (organised or opportunistic screening) in your country? - Yes

^1^ Informants were asked to rate the importance of each barrier that exists in your country in terms of its impact on programme effectiveness and equity

^2^ Informants were asked to select the three most important barriers to overcome

## References

1. Priaulx J, de Koning HJ, de Kok I, et al. Identifying the barriers to effective breast, cervical and colorectal cancer screening in thirty one European countries using the Barriers to Effective Screening Tool (BEST). Health Policy 2018;**122**(11):1190-97.

2. Turnbull E, Priaulx J, van Ravesteyn NT, et al. A health systems approach to identifying barriers to breast cancer screening programmes. Methodology and application in six European countries. Health Policy 2018;**122**(11):1198-205.
