# Supplementary material for: Overcoming barriers: Modelling the effect of potential future changes of organized breast cancer screening in Italy
Source: J Med Screen. 2023 Feb 10;30(3):134–41. doi: 10.1177/09691413231153568 (PMC10399099; doi:10.1177/09691413231153568)
Supplement: sj-docx-2-msc-10.1177_09691413231153568 - Supplemental material for Overcoming barriers: Modelling the effect of potential future changes of organized breast cancer screening in Italy [file sj-docx-2-msc-10.1177_09691413231153568.docx]

**Supplementary Methods**

**EU-TOPIA evaluation tool - User guide**

**– Version January 2020**

**CONTENTS**

[**1.** **Introduction 2**](#_Toc13757113)

[**2.** **MISCAN model 2**](#_Toc13757114)

[**3.** **Account management 3**](#_Toc13757115)

[3.1. Register 3](#_Toc13757116)

[3.2. Log in 3](#_Toc13757117)

[3.3. Log out 3](#_Toc13757118)

[3.4. Account information / password 3](#_Toc13757119)

[3.5. Help 3](#_Toc13757120)

[**4.** **Data collection 4**](#_Toc13757121)

[4.1. Download Excel Data Templates 4](#_Toc13757122)

[4.2. Breast Cancer Tables 6](#_Toc13757123)

[4.3. Essential information for filling out the data tables 7](#_Toc13757124)

[4.4. Country 8](#_Toc13757125)

[*Table0: Country 8*](#_Toc13757126)

[4.5. Epidemiological Data 9](#_Toc13757127)

[*eTable1: Population age composition 9*](#_Toc13757128)

[*eTable2: Breast cancer incidence rates 9*](#_Toc13757129)

[*eTable3: Breast cancer mortality rates 9*](#_Toc13757130)

[*eTable4: Relative survival 10*](#_Toc13757131)

[*eTable5: Breast cancer stage distribution 10*](#_Toc13757132)

[*eTable6: Population all-cause mortality 11*](#_Toc13757133)

[4.6. Screening Data 11](#_Toc13757134)

[*sTable1: Screening strategy 11*](#_Toc13757135)

[*sTable2: Screening coverage 12*](#_Toc13757136)

[*sTable3: Screening history 14*](#_Toc13757137)

[*sTable4: Further assessment indication 15*](#_Toc13757138)

[*sTable5: Further assessment participation 17*](#_Toc13757139)

[*sTable6: Further assessment outcome 18*](#_Toc13757140)

[*sTable7: Outcome 19*](#_Toc13757141)

[*sTable8: Pathological size 20*](#_Toc13757142)

[*sTable9: Surgical treatment 21*](#_Toc13757143)

[*sTable10: Interval cancers 22*](#_Toc13757144)

[*sTable11: Opportunistic screening 23*](#_Toc13757145)

[*sTable 12: Adjuvant treatment 24*](#_Toc13757146)

[**5.** **Simulation 25**](#_Toc13757147)

[5.1. Upload data 25](#_Toc13757148)

[5.2. Quality check of the data 26](#_Toc13757149)

[5.3. Exemplary countries 27](#_Toc13757150)

[5.4. Selection of screening scenarios 27](#_Toc13757151)

[**6.** **Results 29**](#_Toc13757152)

[6.1. Downloading the results 29](#_Toc13757153)

[6.2. Description of the results. 30](#_Toc13757154)

**Introduction**

The overall aim of the EU-TOPIA project is to improve existing cancer screening programmes in Europe. To aid in the improvement of existing programmes, we have developed a web-based evaluation tool based on the MISCAN model to allow European policymakers and researchers to simulate outcomes of multiple cancer screening strategies for their own country. This document describes the structure and the standard process required for utilizing the EU-TOPIA evaluation tool from creating an account in section 3 to analyzing the results of your simulations in section 6.

**MISCAN model**

The EU-TOPIA evaluation tool uses a well-established microsimulation model for cancer (MISCAN-Breast). MISCAN was developed in the 1970s at the Department of Public Health of Erasmus MC, University Medical Center Rotterdam and was designed for evaluating the effect of cancer screening. MISCAN simulates individual life histories and assesses the consequences of introducing a screening programme on these life histories. The model estimates the effect of cancer screening in a dynamic population and can explain results of cancer screening trials and predict and compare the (cost-)effectiveness of different screening strategies. Our model has been calibrated on data from a number of countries exemplary for all European regions

**Account management**

**Register**

First things first: you need to register as a user of EU-TOPIA evaluation tool website.

You can do that by filling out the registration form (<https://miscan.eu-topia.org/registration/register>), click on the link in the confirmation e-mail to confirm your e-mail address and let the EU-TOPIA admins process your information for the final authorization.

**Log in**

Once the EU-TOPIA admins send you an e-mail to confirm your account, it is fully operative. Now you are able to log in into the EU-TOPIA evaluation tool (<https://miscan.eu-topia.org/login>) with your e-mail and password and perform simulations. When you log in the first time, you will be asked to download this user’s guide and to declare to have read it.

**Log out**

You can log out from the application in anytime clicking the button “Logout” at the top right corner of the web site.

**Account information / password**

You can view your account information and/or change your password clicking on “My account” or on your user name (both at the top-right corner of the screen).

**Help**

If you need any help, please feel free to e-mail the EU-TOPIA research team at [eu.topia@erasmusmc.nl](mailto:eu.topia@erasmusmc.nl) or complete the contact form by clicking “Help” at the top-right corner of the screen.

**Data collection**

The EU-TOPIA evaluation tool was designed to allow users to simulate outcomes of several cancer screening strategies for their own country. Thus, this tool requires the users to upload specific demographic and screening data for their own country.

Section **4.1.** describes how to download the excel data templates needed to collect country specific data.

Sections **4.2 to 4.4** describe the excel data templates needed to collect country specific data and give important general information for filling out the data tables. Instructions on how to fill in specific data templates are described in two parts: epidemiological data in section **4.5**, and screening programme related monitoring data in section **4.6**.

**Download Excel Data Templates**

1.
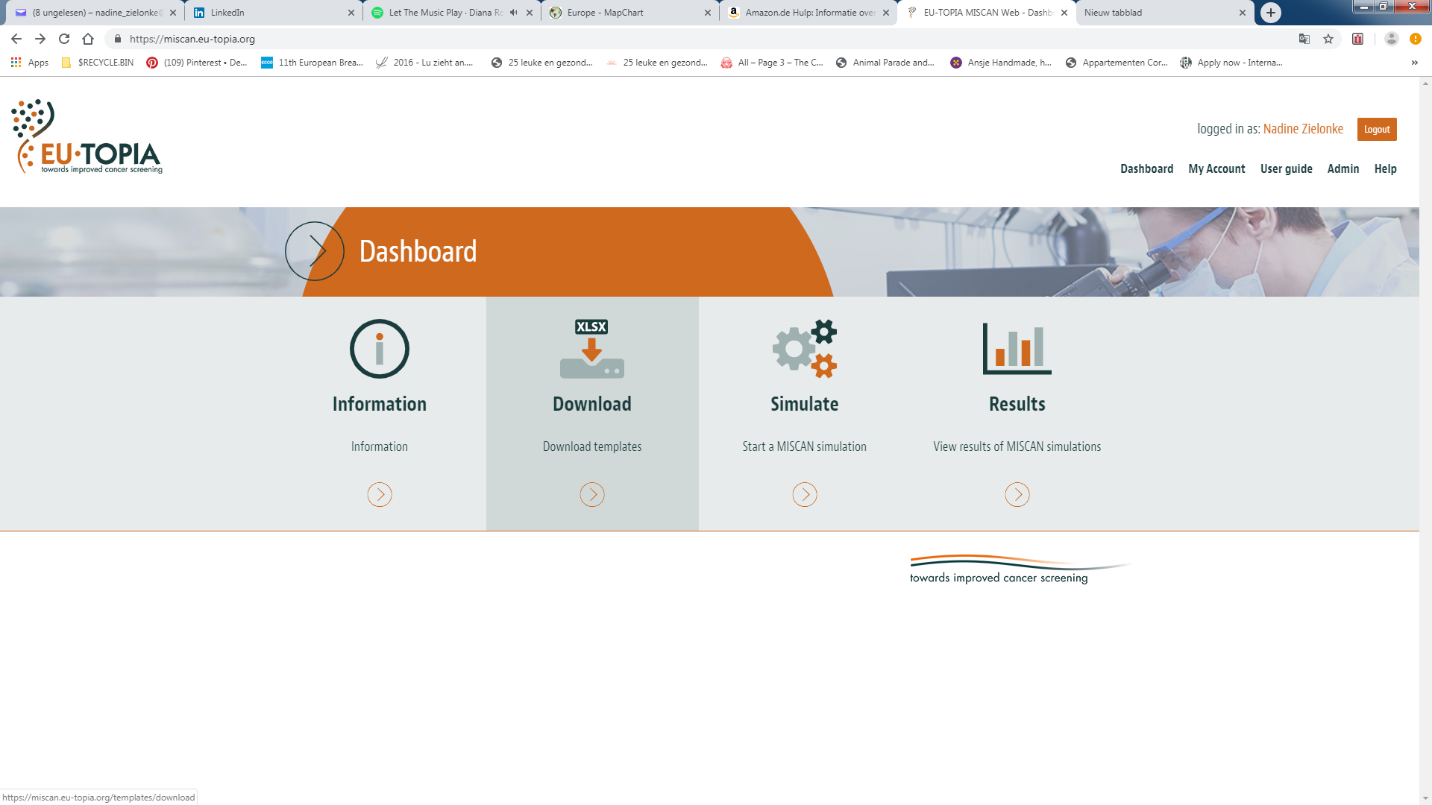
Log on to the main dashboard page.

Once you log in you will see the main evaluation tool dashboard (**Figure 1**).

**Figure 1**. EU-TOPIA evaluation tool, user’s dashboard.

1. Click on “Download”.
2. You can download templates from each cancer site and save these on your computer (see **Figure 2**).

For the breast specific cancer template, click the breast cancer icon.


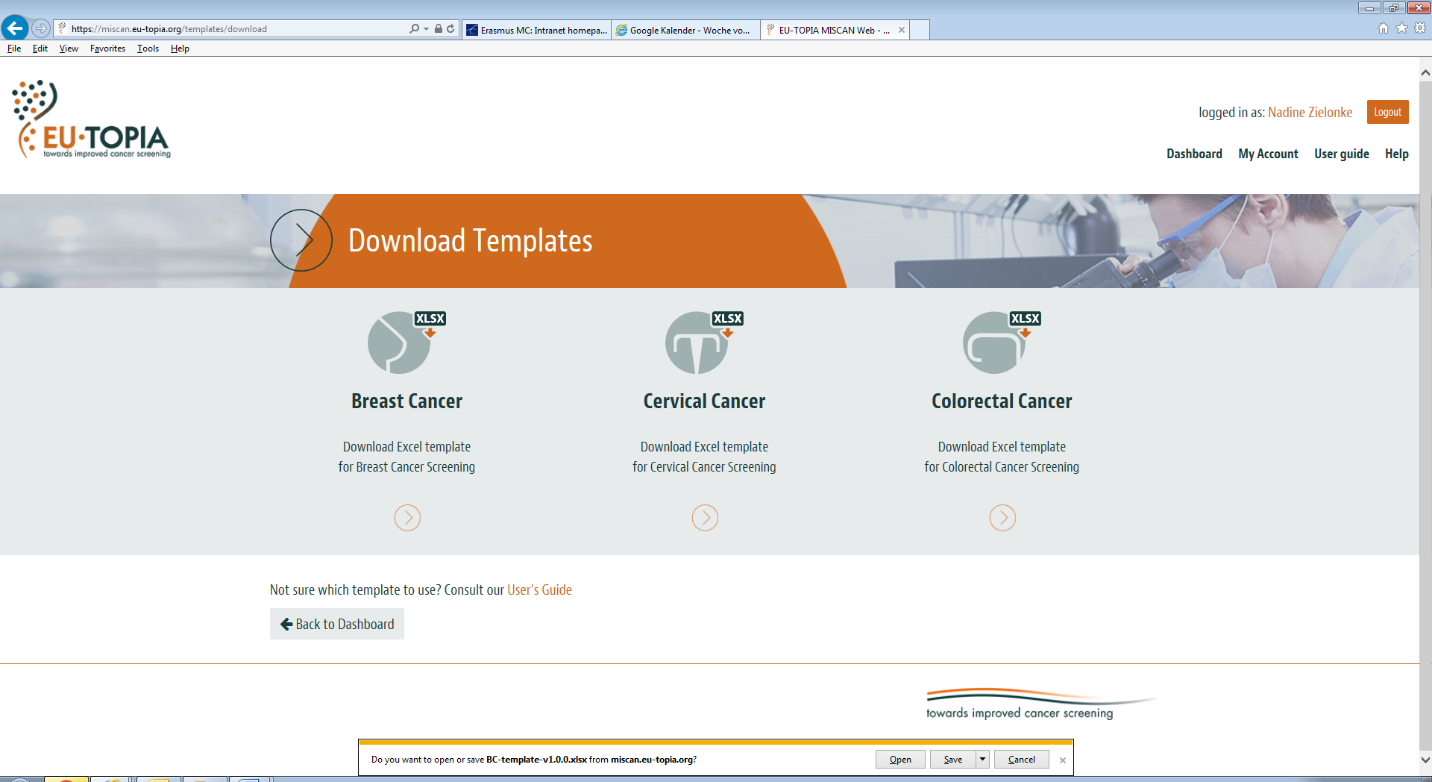
**Figure 2**. EU-TOPIA evaluation tool, download templates section.

1. Save the templates to your computer by clicking “Save as” in Save option list (see **Figure 3**)


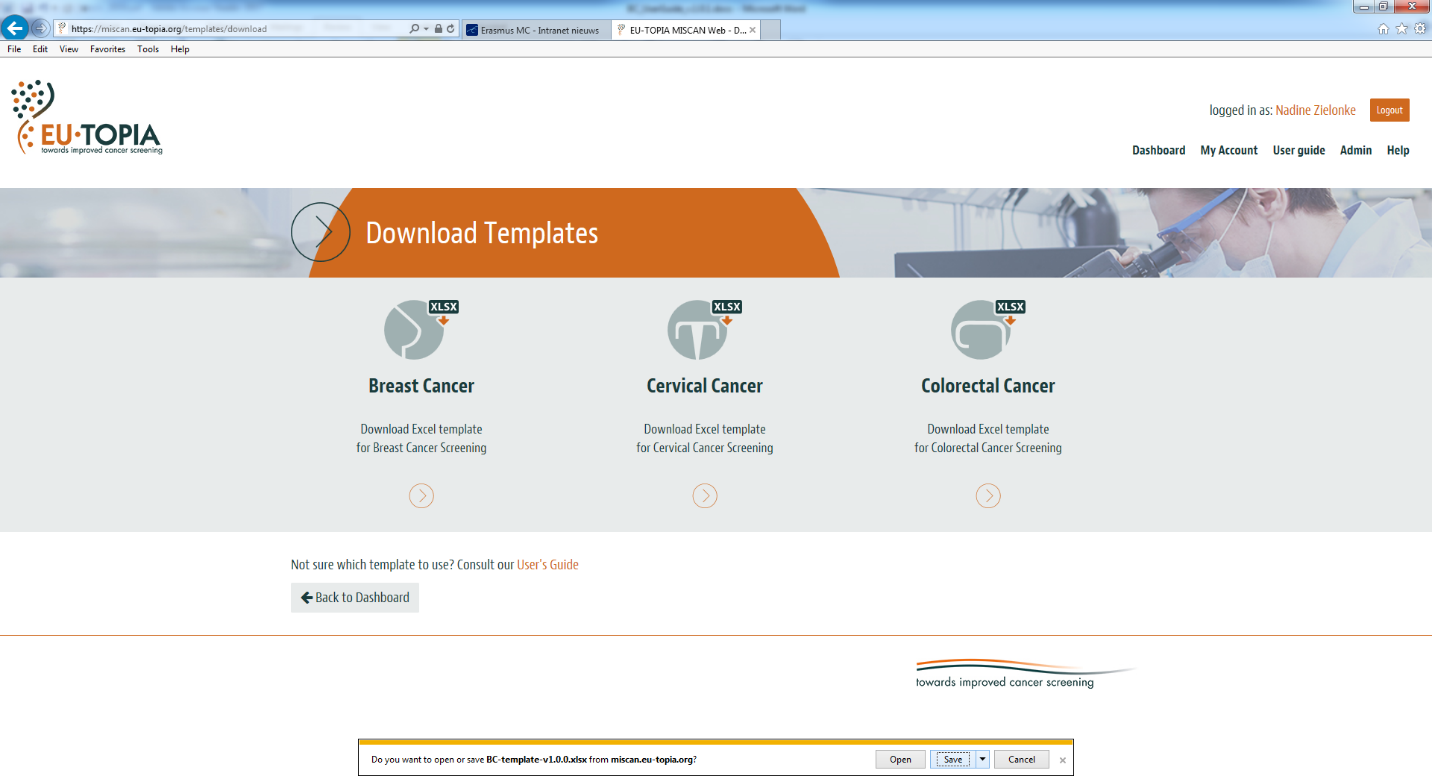
**Figure 3**. EU-TOPIA evaluation tool, saving excel data template.

**Breast Cancer Tables**

In this document you will find instructions on how to fill the Excel tables in order to simulate different BREAST cancer screening strategies for your country.

To tailor the model to your own country, you need to upload the following information:

- population and epidemiological data (6 tables)
- screening monitoring information (12 tables)

A core set of tables is required for the model to run your simulations. These are labelled as ‘mandatory’ in Table 1. Including information labelled as ‘should have’ or ‘nice to have’ will improve the quality of the information the model generates for your country. A more detailed description of each table will be provided from section 3.6.

**Table 1**. Overview of all data requirements for the EU-TOPIA web-based tool for breast cancer.

| **Table name** | **Brief description** | **Level of importance** | **More detailed instructions for filling this table.** |
| --- | --- | --- | --- |
| Table0 | [Select your country](#_Table0:_Country) | Mandatory | [Section 4.4](#_Table0:_Country) |
| eTable1 | [Population data](#_eTable1:_Population_age_1) | Mandatory | [Section 4.5](#_Epidemiological_Data_1) |
| eTable2 | [Cancer incidence](#_eTable2:_Breast_cancer) | Mandatory |  |
| eTable3 | [Cancer mortality](#_eTable3:_Breast_cancer) | Mandatory |  |
| eTable4 | [Relative survival by stage](#_eTable4:_Relative_survival) | Should have |  |
| eTable5 | [Stage distribution](#_eTable5:_Breast_cancer) | Should have |  |
| eTable6 | [All-cause mortality by age](#_eTable6:_Population_all-cause_1) | Should have |  |
| sTable1 | [Screening strategy](#_sTable1:_Screening_strategy) | Mandatory | [Section 4.6](#_Screening_Data) |
| sTable2 | [Screening coverage](#_sTable2:_Screening_coverage) | Mandatory |  |
| sTable3 | [Screening history](#_sTable3:_Screening_history) | Should have |  |
| sTable4 | [Further assessment indication](#_sTable4:_Further_assessment) | Should have |  |
| sTable5 | [Further assessment participation](#_sTable5:_Further_assessment) | Should have |  |
| sTable6 | [Further assessment outcome](#_sTable6:_Further_assessment) | Should have |  |
| sTable7 | [Outcome](#_sTable7:_Outcome) | Should have |  |
| sTable8 | [Pathological sizes](#_sTable8:_Pathological_size) | Nice to have |  |
| sTable9 | [Surgical treatment](#_sTable9:_Surgical_treatment) | Nice to have |  |
| sTable10 | [Interval cancers](#_sTable10:_Interval_cancers) | Should have |  |
| sTable11 | [Opportunistic screening](#_sTable11:_Opportunistic_screening) | Should have |  |
| sTable12 | [Adjuvant treatment](#_sTable_12:_Adjuvant) | Should have |  |

**Essential information for filling out the data tables**

**Key instructions**

- Fill out the tables in the order that they are presented in the excel template.
- ‘Mandatory’ data is necessary to have a good refinement of the model for the country-specific analysis
- The more accurate and complete the country-specific data that you are providing, the more accurate and country-specific will be the results of the simulations.
- Fill only the white cells in the data templates. All other cells are non-editable.
- Only use the data type specified by the information box when you click on an empty cell indicated.
- Be careful with *copying* and *pasting* data from other Excel files. If data is pasted into non-editable cells, the tool will not recognize the inputs.

**Information about data quality**

- After you submit the data, automatic data quality checks will be applied to make sure the inputs fall within a reasonable range.
- The more complete the input data, the better the model will simulate your scenarios.

**Trouble shooting**

- Make sure you always work with the most recent version of the user guide as we are constantly updating this document to answer as many questions as possible.
- If you encounter any problems when entering the data, please contact the EU-TOPIA research team at [eu.topia@erasmusmc.nl](mailto:eu.topia@erasmusmc.nl)

**Organised vs. opportunistic screening**

Based on the IARC Handbook of Cancer Prevention (IARC Working Group. IARC Handbooks of Cancer Prevention, Volume 15: Breast Cancer Screening. Lyon, 2016) we defined

- **organised breast cancer screening** as screening programmes organised at national or regional level, with an explicit policy, specifying age categories, method and interval for screening, a management team responsible for implementation and a health care team for decisions and care; a quality assurance structure and systematic monitoring of quality indicators; a method for monitoring of cancer occurrence in the target population.
  The programme policy includes active invitation of the entire target population and usually also active follow-up of screen-positive subjects (IARC 2005 -2016; and Miles et al. Cancer 2004).
- In contrast, **opportunistic breast cancer screening** refers to mammograms performed as a result of the initiative of women themselves or at time of routine health checks. The classification non-population based (opportunistic) screening applies to areas where individual invitations are not sent to women in the eligible population, or when women undergo a mammography outside, or in addition to, the (existing) screening programme. Registration and monitoring of such screening activity is often unavailable.

**Country**

***Table0: Country***

Select your country from the drop-down menu if you submit national data (=preferred, Step 1 in Figure 1). In that case you can ignore the second drop-down menu (Region). If your country is not listed, please contact us at: [eu.topia@erasmusmc.nl](mailto:eu.topia@erasmusmc.nl)

If you want to submit regional instead of national data, please contact us as at [eu.topia@erasmusmc.nl](mailto:eu.topia@erasmusmc.nl). After we assigned you to an index region, you need to select your country from the first drop-down menu (Step 1 in Figure 1) and the respective index region (A-Z) from the second drop-down menu (Step 2 in **Figure 4**).


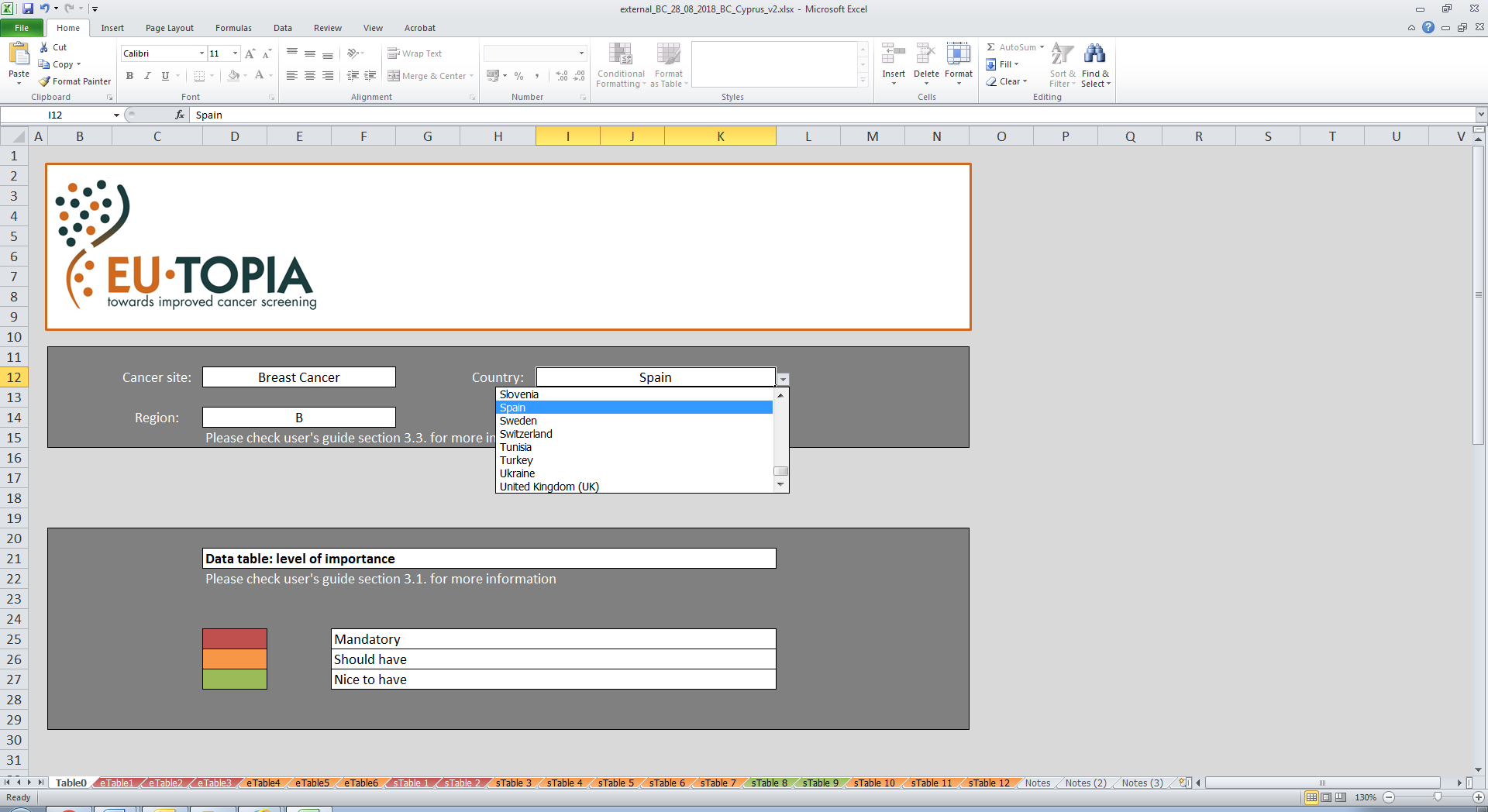


**2**

**1**

**Figure 4**. EU-TOPIA evaluation tool, Select your country (and region) in Table0.

Please be aware that we strongly advise you to use the same reference population in all tables! In case of a mixed situation - where most data is national and some is regional, or vice versa – leave a comment in the email accompanying the submitted data templates.

**Epidemiological Data**

All tables for demographic and epidemiological data are marked as “eTablex”.

Please fill out all of them for your country. In this section, we show you how to fill them out correctly.

***eTable1: Population age composition***

| Level of importance | Mandatory |
| --- | --- |
| What does this table contain? | The 2018 female population and population projections (up to 2050) for your country |
| Format | Separated by calendar year and five-year age groups |
| Potential data sources | National statistical office |
| Potential data sources | We suggest you use the base case scenario (i.e. the scenario based on current population trends). If there are several sources for population data in your country, we suggest using the source which is also used for official government projections. A good resource is EUROSTAT population projections (<http://ec.europa.eu/eurostat/data/database>). |

***eTable2: Breast cancer incidence rates***

| Level of importance | Mandatory |
| --- | --- |
| What does this table contain? | The number of incident breast cancer cases (ICD-10: C50 and D05.1) and person years at risk in 1981-1985 and 2011-2015. |
| Format | Separated by five-year age groups, including Ductal Carcinoma In Situ (DCIS) |
| Potential data sources | National cancer registries |
| Trouble shooting | We prefer the data of 1981-1985 as this represents a period before any form of screening (organised or opportunistic) was introduced in Europe and hence can serve as the background incidence. However, if you know the cancer register was not complete at that time, please fill-out only the table for the period 2011-2015.  For the respective 5-year period, you can either report the sum of cases (diagnoses) and the population, or the average over those five years. Both will be calculated into the incidence rate over that period.  If your estimates are based on small numbers, we recommend using a longer time period in reporting incidence data (10 most recent years instead of 5).  If you do not have direct access to national cancer registry data or detailed data are not available, we suggest checking the availability of cancer incidence data from the IARC cancer in five continents dataset (CI5, <http://ci5.iarc.fr/CI5I-X/Pages/download.aspx>). |

***eTable3: Breast cancer mortality rates***

| Level of importance | Mandatory |
| --- | --- |
| What does this table contain? | Mortality due to breast cancer (ICD-10: C50) in 1981-1985 and 2011-2015. |
| Format | Separated by five-year age groups |
| Potential data sources | National cause of death register |
| Trouble shooting | We prefer the data of 1981-1985 as this represents a period before any form of screening (organised or opportunistic) was introduced in Europe and hence can serve as the background mortality. However, if you know the death register was not complete at that time, please fill-out only the table for the period 2011-2015.  For the respective 5-year period, you can either report the sum of cases (deaths) and the population, ,or the average over those 5 years. Both will be calculated into the incidence rate over that period. |

***eTable4: Relative survival***

| Level of importance | Should have |
| --- | --- |
| What does this table contain? | Probability of surviving 10 years after a diagnosis of Breast cancer (ICD-10: C50), observed in the most recent years |
| What does this table NOT contain? | Carcinoma in situ cases |
| Format | Separated by stage. |
| Potential data sources | National cancer registries |
| Trouble shooting | Tumour (T) of the breast cancer TNM staging system. The staging information should be based on the histopathological assessment of the tumour (pTNM), not the clinical (cTNM). If this information is not available, please leave a comment in the NOTES sheet of the data template.  T describes the size of the tumour (area of cancer).tumour  T1A between 0.1 and 0.5 cm across, independent of node status  T1B between 0.5 cm and 1 cm across, independent of node status  T1C between 1 cm and 2 cm across, independent of node status  T2+ between 2 cm and 5 cm across, independent of node status  Only numbers between 0 and 100 can be used as input (i.e. no words, notes, or symbols): typing 0.2, the web-based tool will read 0.2%; and typing 20, 20%. Survival probabilities that show greater survival in advanced stages compared to lower stages will be marked as “low-quality” data and you will be advised not to use this data when using the web-based tool.  If your estimates are based on small numbers or detailed data are not available, we recommend to check published data from EUROCARE group (<http://www.eurocare.it/>) |

***eTable5: Breast cancer stage distribution***

| Level of importance | Should have |
| --- | --- |
| What does this table contain? | Stage distribution of breast cancer (ICD-10: C50 and D05.1) in 1981-1985 and 2011-2015 |
| Format | Separated by stage Tumour, including Ductal Carcinoma In Situ (DCIS) |
| Potential data sources | National cancer registries |
| Trouble shooting | Tumour (T) of the breast cancer TNM staging system. The staging information should be based on the histopathological assessment of the tumour (pTNM), not the clinical (cTNM). If this information is not available, please leave a comment in the NOTES sheet of the data template.  DCIS Ductal Carcinoma In Situ  T describes the size of the tumour (area of cancer).  T1A between 0.1 and 0.5 cm across, independent of node status  T1B between 0.5 cm and 1 cm across, independent of node status  T1C between 1 cm and 2 cm across, independent of node status  T2+ between 2 cm and 5 cm across, independent of node status  If your stage distribution adds up to more than 100%, the web-based tool will mark your data as “low-quality” data (additional function in the web-based tool). In that case please contact the EU-TOPIA research group at: [eu.topia@erasmusmc.nl](mailto:eu.topia@erasmusmc.nl) |

***eTable6: Population all-cause mortality***

| Level of importance | Should have |
| --- | --- |
| What does this table contain? | Current all-cause mortality rate for women |
| Format | By single ages (0-100) |
| Potential data sources | Life tables from national statistical offices |
| Trouble shooting | You are only allowed to input numbers (no words, notes, or symbols) between 0 and 1. Please make sure that values are not multiplied by 100,000 person-years (hence, values need to be reported considering 1 person-year).  The inputs need to be age-specific mortality rates, so no age-specific probabilities of death. In the projections of the web-based tool, the all-cause mortality will be assumed to stable over time.  As an alternative data source, we recommend your country’s life tables from the Human Mortality database (<http://www.mortality.org/>, use the ‘mx’ column in the data provided). |

**Screening Data**

All tables for screening data are marked as “sTablex”.

Please fill out all of them for your country. In this section, we show you how to fill them out correctly.

***sTable1: Screening strategy***

| Level of importance | | Mandatory |
| --- | --- | --- |
| What does this table contain? | | A summary of the current screening strategy of your country |
| What does this table NOT contain? | | Data on opportunistic screening |
| Potential data sources | | National breast cancer screening programme |
| Format | | Separated by five-year age groups |
| **Detailed content description** | | |
| Country (or Region): | | Enter the country or area to which all tables refer. |
| Index year | | All tables should report data from that index year |
| Starting year of the programme | | When was the organised breast cancer screening programme introduced in your country/region? |
| End of the roll-out phase | | Enter the respective year in case the roll-out was completed before the index year. |
| A1 | Target population | Total number of age-eligible women obtained from official statistics (irrespective of the screening interval). |
| A2 | Screening interval | Interval (in years) between routine screens decided upon in each screening programme dependent on the screening practice in your country. |
| Trouble shooting | | As index year you should pick the most recent year you have complete (follow up) data on. Note that in sTable2 data will be required up to June of the following year.  If screening is not implemented uniformly across the country or region on which you are reporting (i.e. there is regional variation in the rollout of screening or there is regional variation in the eligible age range or frequency) please report a screening policy which best represents the most common policy in your country.  Consider for example a country where some regions invite women each year between age 45 and 49 and every two years between 50 and 69, whereas other regions only invite women between 50 and 74 biannually. Given the population sizes and the representativeness of the regions, you can either report only one of them as the screening strategy of your country. Alternatively, you can report a joined version of those two screening policies.  We propose you enter a screening frequency that best reflects the screening practice in your country, i.e. if you invite women more often than every 24 months, you could enter e.g. 1.8 years. When you chose the screening scenarios (see section [5.4](#_Selection_of_screening)), you can simulate the effect of an optimal frequency of e.g. 2 years compared to the over- or underscreening currently present in your country.  It is possible to indicate different screening intervals per age group. However, the model calculations are solely based on the frequency entered at age-group 50-54.  If you are in doubt please contact us at: [eu.topia@erasmusmc.nl](mailto:eu.topia@erasmusmc.nl)  If your data cannot be stratified by five-year age groups, put the total amount in the row marked as “unknown”.  In a mixed situation, with data from some areas which can be stratified and other data that cannot be stratified, please fill separately the first rows for the former and the last row for the latter.  Always check the total figures at the bottom of each table to be sure that the sum of the strata is the total number expected. |

***sTable2: Screening coverage***

| Level of importance | | Mandatory |
| --- | --- | --- |
| What does this table contain? | | Screening invitations and screening tests |
| What does this table NOT contain? | | Data on opportunistic screening |
| Potential data sources | | National breast cancer screening programme |
| Format | | Absolute number, separated by five-year age groups |
| **Detailed content description** | | |
| B1 | Individuals personally invited in index year | Enter the number of all personally invited women (not counting reminders or returned letters) in the period to which data refer (from January 1st to December 31st).  Do not include invitations to intermediate mammograms (short term recalls) in this column. |
| B2 | Individuals screened of invited in index year | This is a subset of B1. Enter the number of women who received a test – counting any test performed up to June of the following year (invitation cohort*).  It is also acceptable, assuming steady state, to estimate this number using the number of attendees in the index year - regardless of their invitation date.  Do not include tests referring to intermediate mammograms (short term recalls) in this column. |
| B3 | Individuals screened in index year | Women who received a test in the index year – regardless of when invited (examination cohort**).  Do not include tests referring to intermediate mammograms (short term recalls) in this column. |
| Trouble shooting | | If your data cannot be stratified by five-year age groups, put the total amount in the row marked as “unknown”. In a mixed situation, with data from some areas which can be stratified and other data that cannot be stratified, please fill separately the first rows for the former and the last row for the latter.  Always check the total figures at the bottom of each table to be sure that the sum of the strata is the total number expected. |

**Invitation cohort:* It includes women attending screening until June 30^th^ of the year following the reference one. It can be used to estimate the response rate (leaving at least 6-month interval available for responding, to the first invitation or to the reminder for women invited at the end of the reference year). Complete data about screening outcomes (including results of assessment and treatment, if indicated) might not be available for women in this cohort until the end of the year following the reference one. Therefore, to obtain accurate measures of screening performance it might be more efficient to refer to the examination cohort

***Examination cohort:* It includes women screened during the reference year, independent of the invitation date. For women in this cohort the information about screening results is generally available by June 30^th^ of the year following the reference one, which allows to get meaningful data about quality indicators within a reasonable interval.

You can decide, based on the availability of the data about outcomes, to use either the invitation or the examination cohort as the denominator for calculating screening performance indicators (sTables 4 to 7).

***sTable3: Screening history***

| Level of importance | | Should have |
| --- | --- | --- |
| What does this table contain? | | Screening invitations and screening tests by screening history |
| What does this table NOT contain? | | Data on opportunistic screening |
| Potential data sources | | National breast cancer screening programme |
| Format | | Absolute numbers, separated by five-year age groups  Data should be stratified per initial/subsequent tests:   - **Initial screening** is the first screening examination of individual women within the screening programme, regardless of the organisational screening round in which the examination takes place. Include also screening tests performed in a population-based screening programme before receiving the first invitation (these examinations are often referred to as “spontaneous tests”). - **Subsequent screening** includes all screening examinations of individual women within the screening programme following an initial screening examination, regardless of the organisational screening round in which the examination takes place. - **Unknown if initial or subsequent** strata should be used for tests for which the above distinction is not available.   The numbers collected in the three sub-tables should refer to strictly distinct sets of women. Always check the total figures at the bottom of the three tables to be sure that the sum of the strata is the total number expected. |
| **Detailed content description** | | |
| C1-C4 | Individuals personally invited in index year | It includes all personally invited women (not counting reminders or returned letters) in the period to which data refer.  C1: women receiving their first invitation in the programme  C2: women invited in the reference year who had already been invited in previous screening rounds  C3 (sub-set of C2): women invited in the reference year who had been invited in previous screening rounds and had not attended the last invitation  C4 (sub-set of C2): women invited in the reference year who had been invited in previous screening rounds and had attended the last invitation  Please indicate the number of women invited from January 1^st^ to December 31^st^ of the index year. Do not include invitations to intermediate mammograms (short term recalls) in these columns. |
| C5-C8 | Individuals screened of invited in index year | It is a subset of the women-invited-in-index-year (C1) who received a test – counting any test performed up to June of the following year (Invitation cohort). It is also acceptable, assuming steady state, to estimate this number using the number of attenders in the index year - regardless of their invitation date (examination cohort).  Do not include tests referring to intermediate mammograms (short term recalls) in these columns.  C5: women receiving their first test in the programme  C6: women who had already received previous test in the programme  C7 (sub-set of C6): women who had received previous tests in the programme, but who had not attended in the previous round  C8 (sub-set of C6): women who had received previous tests in the programme and had attended in the previous round. |
| C9-C12 | Individuals screened in index year | Women who received a test in index year – regardless of when invited. Do not include tests referring to intermediate mammograms (short term recalls) in these columns  C9: women receiving their first test in the programme  C10: women who had already received previous test in the programme  C11 (Sub-set of C10): women who had received previous tests in the programme, but who had not attended in the previous round  C12 (Sub-set of C10): women who had received previous tests in the programme and had attended in the previous round. |
| Trouble shooting | | If your data cannot be stratified by five-year age groups, put the total amount in the row marked as “unknown”. In a mixed situation, with data from some areas which can be stratified and other data that cannot be stratified, please fill separately the first rows for the former and the last row for the latter.  Always check the total figures at the bottom of each table to be sure that the sum of the strata is the total number expected. |

***sTable4: Further assessment indication***

| Level of importance | | Should have |
| --- | --- | --- |
| What does this table contain? | | Further assessment as an additional diagnostic technique (either at screening or at recall) that are performed for medical reasons in order to clarify the nature of a perceived abnormality detected at the screening examination.  It may include breast clinical examination, additional imaging and invasive investigations (cytology, core biopsy). Further assessment may have taken place on the same day as the screening examination or on recall. Please include among positive women also those undergoing assessment (as defined) on the same day as screening and not only those referred for assessment at a different date/session |
| What does this table NOT contain? | | Data on breast cancer detected by opportunistic screening |
| Potential data sources | | National breast cancer screening programme |
| Format | | Absolute numbers, separated by five-year age groups  Data should be stratified per initial/subsequent tests:   - **Initial screening** is the first screening examination of individual women within the screening programme, regardless of the organisational screening round in which the examination takes place. Include also screening tests performed in a population-based screening programme before receiving the first invitation (these examinations are often referred to as “spontaneous tests”). - **Subsequent screening** includes all screening examinations of individual women within the screening programme following an initial screening examination, regardless of the organisational screening round in which the examination takes place. - **Unknown if initial or subsequent** strata should be used for tests for which the above distinction is not available.   The numbers collected in the three sub-tables should refer to strictly distinct sets of women. Always check the total figures at the bottom of the three tables to be sure that the sum of the strata is the total number expected. |
| **Detailed content description** | | |
| D1 | Individuals screened in index year | In this column you should report the number of women screened in the reference year : i.e. sTable2 column B3, if examination cohort, or column B2, if invitation cohort.  This column refers to the denominator of the “Recall rate” indicator, i.e. if the numerator (number of further assessment recommended) has not been provided by all areas, then report the number of women screened in the areas where data on the number of further assessment recommendation are available. |
| D2 | Positive | Women who have been recommended further assessment (it is a subset of D1). |
| D3 | Negative | Women who have not been recommended further assessment (it is a subset of D1). |
| Trouble shooting | | If your data cannot be stratified by five-year age groups, put the total amount in the row marked as “unknown”. In a mixed situation, with data from some areas which can be stratified and other data that cannot be stratified, please fill separately the first rows for the former and the last row for the latter.  Always check the total figures at the bottom of each table to be sure that the sum of the strata is the total number expected.  Consider for example in a country where:  - 20 regions provide relevant information for calculating compliance  - 15 of these regions have data on recall  In this case:  - the number of women screened documented in table 2 will refer to the 20 regions  - the number of women screened documented in table 3 will refer to the 15 regions. |

***sTable5: Further assessment participation***

| Level of importance | | Should have |
| --- | --- | --- |
| What does this table contain? | | Further assessment participation among positive women. |
| What does this table NOT contain? | | Data on opportunistic screening |
| Potential data sources | | National breast cancer screening programme |
| Format | | Absolute numbers, separated by five-year age groups. Each woman is counted only once.  Data should be stratified per initial/subsequent tests:   - **Initial screening** is the first screening examination of individual women within the screening programme, regardless of the organisational screening round in which the examination takes place. Include also screening tests performed in a population-based screening programme before receiving the first invitation (these examinations are often referred to as “spontaneous tests”). - **Subsequent screening** includes all screening examinations of individual women within the screening programme following an initial screening examination, regardless of the organisational screening round in which the examination takes place. - **Unknown if initial or subsequent** strata should be used for tests for which the above distinction is not available.   The numbers collected in the three sub-tables should refer to strictly distinct sets of women. Always check the total figures at the bottom of the three tables to be sure that the sum of the strata is the total number expected. |
| **Detailed content description** | | |
| E1 | Positive | By default, the column will report the number of the women who have been recommended further assessment, column D2 in sTable4. Hence it refers to the denominator of the "Further assessment participation rate" indicator, so if the numerator (number of further assessment performed) has not been provided by all areas, then report the number of positive women in the areas where data on number of further assessment performance are available. |
| E2 | Further assessment performed | Women who actually underwent further imaging and/or invasive assessment, irrespective of whether further assessment was complete or not (it is a subset of E1). |
| E3 | Further assessment not performed | Women who did not undergo further assessment (it is a subset of E1). |
| Trouble shooting | | If your data cannot be stratified by five-year age groups, put the total amount in the row marked as “unknown”. In a mixed situation, with data from some areas which can be stratified and other data that cannot be stratified, please fill separately the first rows for the former and the last row for the latter.  Always check the total figures at the bottom of each table to be sure that the sum of the strata is the total number expected. |

***sTable6: Further assessment outcome***

| Level of importance | | Should have |
| --- | --- | --- |
| What does this table contain? | | Results of further assessment |
| What does this table NOT contain? | | Data on opportunistic screening |
| Potential data sources | | National breast cancer screening programme |
| Format | | Absolute numbers, separated by five-year age groups. Each woman is counted only once.  Data should be stratified per initial/subsequent tests:   - **Initial screening** is the first screening examination of individual women within the screening programme, regardless of the organisational screening round in which the examination takes place. Include also screening tests performed in a population-based screening programme before receiving the first invitation (these examinations are often referred to as “spontaneous tests”). - **Subsequent screening** includes all screening examinations of individual women within the screening programme following an initial screening examination, regardless of the organisational screening round in which the examination takes place. - **Unknown if initial or subsequent** strata should be used for tests for which the above distinction is not available.   The numbers collected in the three sub-tables should refer to strictly distinct sets of women. Always check the total figures at the bottom of the three tables to be sure that the sum of the strata is the total number expected. |
| **Detailed content description** | | |
| F1 | Individuals screened of invited in index year | By default, the column will report the number of women screened in the reference year, you reported in column D1, sTable4. Hence, it is also the denominator of the "Surgical referral rate" indicator.  If the numerator (number of further assessment recommended) has not been provided by all areas, then report the number of women screened in the areas where data on surgical referral are available. |
| F2 | Further assessment performed | By default, the column will report the number of women who underwent further imaging and/or invasive assessment, column E2 in sTable5.  In the programme or areas where data is available on treatment referral, these are the women who actually underwent imaging and/or invasive further assessment, irrespective of whether further assessment was complete or not. |
| F3 | Treatment/Surgery referral or inoperable ca | Women referred to open surgical biopsy or surgical intervention or neo-adjuvant therapy as a result of assessment, including also those with cancers that are not fit for surgery or other treatment (it is a subset of F2). |
| F4 | Negative | This includes all other possible known results of assessment (it is a subset of F2). Please include also "Short Term recall", being a mammogram performed out of sequence with the screening interval (say at 6 or 12 months for programme with two-years screening interval), as a result of the screening test (not recommended by the European Guidelines) or as a result of further assessment. |
| Trouble shooting | | If your data cannot be stratified by five-year age groups, put the total amount in the row marked as “unknown”.  In a mixed situation, with data from some areas which can be stratified and other data that cannot be stratified, please fill separately the first rows for the former and the last row for the latter.  Always check the total figures at the bottom of each table to be sure that the sum of the strata is the total number expected. |

***sTable7: Outcome***

| Level of importance | | Should have |
| --- | --- | --- |
| What does this table contain? | | Screening outcomes.  Please indicate the most advanced lesion per woman. |
| What does this table NOT contain? | | Data on opportunistic screening |
| Potential data sources | | National breast cancer screening programme |
| Format | | Absolute numbers, separated by five-year age groups.  Each woman is counted only once.  Data should be stratified per Initial/subsequent tests:   - **Initial screening** is the first screening examination of individual women within the screening programme, regardless of the organisational screening round in which the examination takes place. Include also screening tests performed in a population-based screening programme before receiving the first invitation (these examinations are often referred to as “spontaneous tests”). - **Subsequent screening** includes all screening examinations of individual women within the screening programme following an initial screening examination, regardless of the organisational screening round in which the examination takes place. - **Unknown if initial or subsequent** strata should be used for tests for which the above distinction is not available.   The numbers collected in the three sub-tables should refer to strictly distinct sets of women. Always check the total figures at the bottom of the three tables to be sure that the sum of the strata is the total number expected. |
| **Detailed content description** | | |
| G1 | Individuals screened of invited in index year | This column refers to column D1 in sTable4, which is also the denominator of the "Surgical referral rate" indicator, calculated in sTable6.  If the numerator (number of further assessment recommended) has not been provided by all areas, then report the number of women screened in the areas where data on surgical referral are available. |
| G2 | Further assessment performed | By default, the column will report the number of women screened in the reference year, column E2 in sTable5.  In the programmes or areas where data is available on treatment referral, these are the women who actually underwent imaging and/or invasive further assessment, irrespective of whether further assessment was complete or not. |
| G3 | Benign lesions, or no lesion | Negative screening test result |
| G4 | CIS | Women with in situ carcinoma detected (ductal or lobular). |
| G5 | Invasive breast cancer | Screen detected invasive cancers (any stage/pathological size) |
| G6 | Other histology | Inflammatory node; non epithelial cancer |
| Trouble shooting | | Screen detection means that the diagnostic assessment process following a positive primary screening examination has been completed. This process should usually be finished within six month. However, if organizational characteristics or constrains require a longer period, include all cancers diagnosed through screening in the period that suit your programme best .  If your data cannot be stratified by five-year age groups, put the total amount in the row marked as “unknown”. In a mixed situation, with data from some areas which can be stratified and other data that cannot be stratified, please fill separately the first rows for the former and the last row for the latter.  Always check the total figures at the bottom of each table to be sure that the sum of the strata is the total number expected. |

***sTable8: Pathological size***

| Level of importance | | Nice to have |
| --- | --- | --- |
| What does this table contain? | | Pathological size of screen detected INVASIVE cancers.  Please indicate the most advanced lesion per woman. |
| What does this table NOT contain? | | Data on opportunistic screening |
| Potential data sources | | National breast cancer screening programme |
| Format | | Tumour (T) of the breast cancer TNM staging system. The staging information should be based on the histopathological assessment of the tumour (pTNM), not the clinical (cTNM). If this information is not available, please leave a comment in the NOTES sheet of the data template.  T describes the size of the tumour (area of cancer)  Absolute numbers, separated by five-year age groups.  Each woman is counted only once.  Data should be stratified per Initial/subsequent tests:   - **Initial screening** is the first screening examination of individual women within the screening programme, regardless of the organisational screening round in which the examination takes place. Include also screening tests performed in a population-based screening programme before receiving the first invitation (these examinations are often referred to as “spontaneous tests”). - **Subsequent screening** includes all screening examinations of individual women within the screening programme following an initial screening examination, regardless of the organisational screening round in which the examination takes place. - **Unknown if initial or subsequent** strata should be used for tests for which the above distinction is not available.   Screen detection means that the diagnostic assessment process following a positive primary screening examination has been completed. This process should usually be finished within six month. However, if organizational characteristics or constrains require a longer period, include all cancers diagnosed through screening in the period that suits your programme best .  The numbers collected in the three sub-tables should refer to strictly distinct sets of women. Always check the total figures at the bottom of the three tables to be sure that the sum of the strata is the total number expected. |
| **Detailed content description** | | |
| I1 | T1A | Tumours between 0.1 and 0.5 cm across, independent of node status |
| I2 | T1B | Tumours between 0.5 cm and 1 cm across, independent of node status |
| I3 | T1C | Tumours between 1 cm and 2 cm across, independent of node status |
| I4 | T2-T4 | Tumours between 2 cm and 5 cm across, independent of node status |
| Trouble shooting | | If your data is available in a different staging system than TNM, please consider the indication for the tumour size only and fill the columns based on that information.  If your data cannot be stratified by five-year age groups, put the total amount in the row marked as “unknown”. In a mixed situation, with data from some areas which can be stratified and other data that cannot be stratified, please fill separately the first rows for the former and the last row for the latter.  Always check the total figures at the bottom of each table to be sure that the sum of the strata is the total number expected. |

***sTable9: Surgical treatment***

| Level of importance | Nice to have |
| --- | --- |
| What does this table contain? | Treatment of cancer |
| What does this table NOT contain? | Data on opportunistic screening |
| Potential data sources | National breast cancer screening programme |
| Format | Surgical treatment, separated by age groups.  Each woman is counted only once.  Data should be stratified as follows:   - Screen detected in situ carcinoma (ductal or lobular). - Screen detected invasive carcinomas   Data should be stratified according to the final surgery, for example in cases when breast conservation therapy is followed by mastectomy, the final surgery is mastectomy. |
| **Detailed content description** | |
| Trouble shooting | Only proportions larger than 0% can be entered.  Screen detection means that the diagnostic assessment process following a positive primary screening examination has been completed. This process should usually be finished within six month. However, if organizational characteristics or constrains require a longer period, include all cancers diagnosed through screening in the period that suit your programme best .  If your data cannot be stratified by a, put the total amount per treatment in the row marked as “unknown”. In a mixed situation, with data from some areas which can be stratified and other data that cannot be stratified, please fill separately the first rows for the former and the last row for the latter. |

***sTable10: Interval cancers***

| Level of importance | | Should have |
| --- | --- | --- |
| What does this table contain? | | Interval cancers |
| What does this table NOT contain? | | Data on opportunistic screening |
| Potential data sources | | National breast cancer screening programme |
| Format | | Absolute numbers, separated by five-year age groups.  Each woman is counted only once.  Data should be stratified per Initial/subsequent tests:   - **Initial screening** is the first screening examination of individual women within the screening programme, regardless of the organisational screening round in which the examination takes place. Include also screening tests performed in a population-based screening programme before receiving the first invitation (these examinations are often referred to as “spontaneous tests”). - **Subsequent screening** includes all screening examinations of individual women within the screening programme following an initial screening examination, regardless of the organisational screening round in which the examination takes place. - **Unknown if initial or subsequent** strata should be used for tests for which the above distinction is not available.   The numbers collected in the three sub-tables should refer to strictly distinct sets of women. Always check the total figures at the bottom of the three tables to be sure that the sum of the strata is the total number expected. |
| **Detailed content description** | | |
| M1 | Interval cancers diagnosed within the first year after test | Number of women diagnosed with an interval cancer within 12 months since the last negative mammography |
| M2 | Interval cancers diagnosed within the second year after test | Number of women diagnosed with an interval cancer between 12 and 24 months since the last negative mammography |
| M3 | Interval cancers diagnosed within the third year after test | Number of women diagnosed with an interval cancer between 24 and 36 months since the last negative mammography.  This only applies to countries with a three-year interval programme. In a two-year programme, column M3 will be empty. |
| M4 | Last negative test in (reference year) - 2 | Number of women with a negative primary screen and the number of negative further assessment performed 2 years before the reference year for data collection (i.e. previous screening round) |
| Trouble shooting | | If the index year for your reported data is 2016, M1 should report on women screened in 2015 and diagnosed with an interval cancer in 2016, whereas M2 reports on women screened in 2014 and diagnosed with an interval cancer in 2016. M4 is the number of negative primary screens plus the number of negative further assessment in 2014 (two years prior to the index year)  Interval cancers can occur in two scenarios:  Women A: negative screen – breast cancer clinically detected after XX months 🡪 interval cancer  Women B: positive screen – negative further assessment – breast cancer clinically detected after XX months 🡪 interval cancer  Both interval cancers, A and B, should be counted here and would set the numerator of the interval cancer rate, whereas M3 is the denominator.  If your data cannot be stratified by five-year age groups, put the total amount in the row marked as “unknown”. In a mixed situation, with data from some areas which can be stratified and other data that cannot be stratified, please fill separately the first rows for the former and the last row for the latter.  Always check the total figures at the bottom of each table to be sure that the sum of the strata is the total number expected. |

***sTable11: Opportunistic screening***

| Level of importance | Should have |
| --- | --- |
| What does this table contain? | An estimate of the proportion of women (of all women in the population) who underwent a mammography outside the organised programme in the past two years. |
| What does this table NOT contain? | Data on population-based, organised screening |
| Potential data sources | National surveys, National social insurance institution |
| Format | Separated by five-year age groups. |
| Trouble shooting | The classification non-population based (opportunistic) screening applies to areas where individual invitations are not sent to the women in the eligible population or when women undergo a mammography outside or additionally to the (existing) screening programme.  Together with sTable2, this information enables the EU-TOPIA team to include the TOTAL screening reality into the simulation of your country or region.  As an example of a mixed situation, think of women who have been invited to the organised screening programme three years ago, who did not follow the invitation but got screened opportunistically in the index year instead. Those women have to be counted in this table.  If you have individual screening histories but you are unable to disentangle them into “organised” and “opportunistic” correctly, then the available data should be put into the respective tables for organised screening only (sTable2).  If your data cannot be stratified by five-year age groups, put the total amount in the row marked as “unknown”. |

***sTable 12: Adjuvant treatment***

| Level of importance | Should have |
| --- | --- |
| What does this table contain? | Adjuvant treatment of all cancers (detected inside or outside a screening programme) |
| What does this table NOT contain? | Surgical treatment and radiology |
| Potential data sources | National breast cancer screening programme, hospital or insurance data |
| Format | Proportions of adjuvant treatment, separated by age groups.  The data reported here should refer to all cancer cases detected in the index year plus the year prior to that (e.g. if your index year is 2016, the cancers you report should have been diagnosed in either 2015 or 2016), independent of when or whether the woman has ever been screened.  Each woman is counted only once.  Data should be stratified per cancer stage and by therapy   - **CIS**: in situ carcinoma (ductal or lobular) - **T1a/b:** tumours between 0.1 and 1 cm across, independent of node status - **T1c:** tumours between 1 cm and 2 cm across, independent of node status - **T2-T4:** Node positive tumours between 2 cm and 5 cm across, independent of node status - No adjuvant therapy: no other treatment except for surgery or radiation - Chemotherapy: adjuvant to surgery and/or radiation - Hormonal therapy: adjuvant to surgery and/or radiation - Combined: a combination of chemotherapy and hormonal therapy |

**Simulation**

In this section you will find instructions on how to use the EU-TOPIA evaluation tool to simulate different breast cancer screening strategies for your country.

After registering and filling out the Excel data templates, as described in section 3, you are ready to start a simulation.

**Upload data**

1. Log in into the EU-TOPIA evaluation tool (<https://miscan.eu-topia.org/login>) with your e-mail and password.
2. Go to the simulation section in the MISCAN web- tool dashboard.
3. Start by selecting a cancer type, breast cancer in this case, and giving your simulation a name. (**Figure 5**).
4. Then, upload the data for your country by clicking “Choose file” and select the data template that you completed as described in Section 4.

Users from one of the exemplary countries (Finland, Italy, the Netherlands and Slovenia) can click on the “Choose” button and either directly select the data from their country in the drop-down menu or select “upload your own data” to upload the data template.


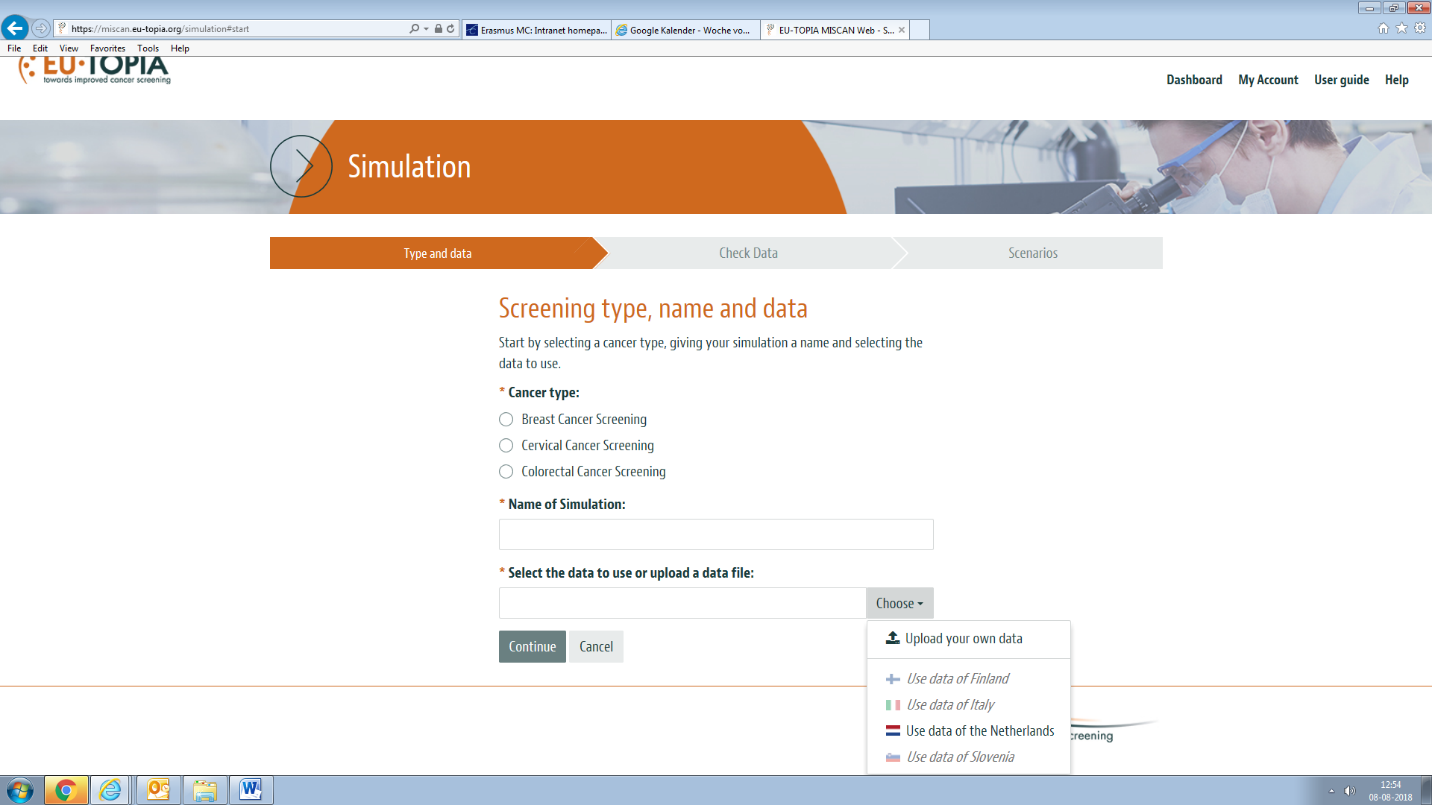
Press “Continue” (**Figure 5**).

**Figure 5**. EU-TOPIA evaluation tool, Screening type, name and data

**Quality check of the data**

After uploading your data, the web-based tool performs checks on data quality and completeness.

In the next step you are asked to verify your data (**Figure 6**). If you wish you can choose to use data already provided by the exemplary country of your region (North: Finland, South: Italy, West: the Netherlands, East: Slovenia) for non-mandatory data. If the data that you uploaded is missing (indicated by a red cross), incomplete or of insufficient quality (indicated by an orange cross) for specific non-mandatory tables, the tool will automatically use the respective data from the exemplary country.

If you did enter data in the template but the evaluation tool marks your data as of insufficient quality, please check if the format of your data is as specified in section 4, within the allowed limits and proportions add up to 100%, if applicable. Also, read the ‘trouble shooting’ part at the bottom of the respective instruction table in section 3 if present. If you still encounter problems with data quality, please contact us at [eu.topia@erasmusmc.nl](mailto:eu.topia@erasmusmc.nl)

Press “Continue” (**Figure 6**).


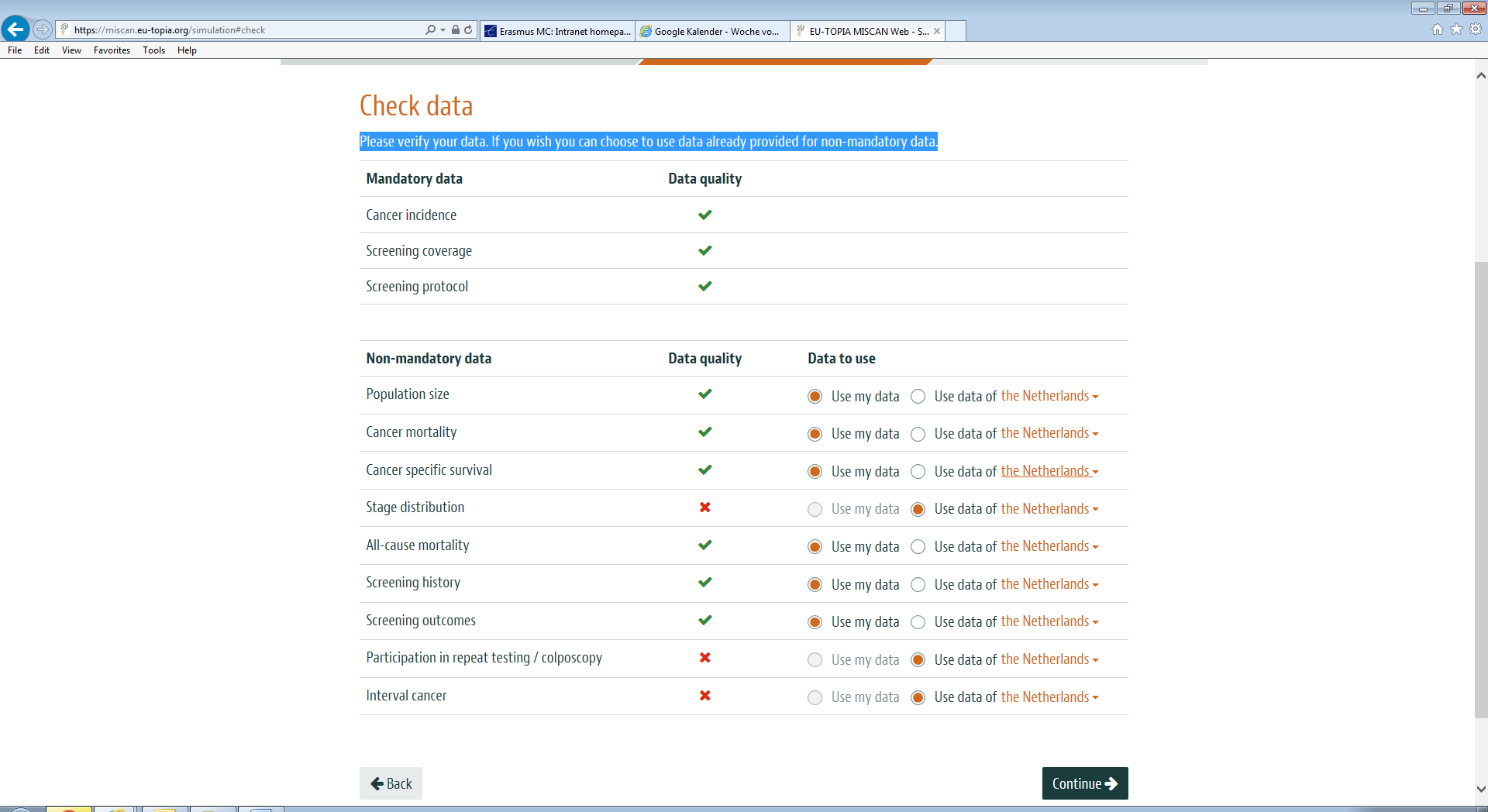


**Figure 6**. EU-TOPIA evaluation tool, verify your data

**Exemplary countries**

Based on the country that you registered from, the EU-TOPIA admins will assign you to one out of four European regions (see list below). Based on that region, model parameters from the exemplary country of that region are used in case non-mandatory data is missing (Figure 6). Also, the parameters that were calibrated as described in the model description (available via the “information” icon at the dashboard), are based on the regional model of the exemplary country. Background information on all of the four exemplary countries is also available via the “information” icon at the dashboard in the fact sheets.

If you think that your country would be better represented by one of the other three exemplary countries, you can consult the EU-TOPIA research team at [eu.topia@erasmusmc.nl](mailto:eu.topia@erasmusmc.nl)

**Northern Europe:** Finland (exemplary country), Denmark, Estonia, Faroe Islands, Iceland, Latvia, Lithuania, Norway and Sweden.

**Southern Europe:** Italy (exemplary country), Cyprus, Gibraltar, Greece, Malta, Portugal and Spain.

**Eastern Europe:** Slovenia (exemplary country), Bulgaria, Czech Republic, Croatia, Hungary, Poland, Romania and Slovakia.

**Western Europe:** The Netherlands (exemplary country), Austria, Belgium, France, Germany, Ireland, Luxembourg, United Kingdom and Switzerland.

**Selection of screening scenarios**

Now you are asked to define the settings of each scenarios you want to simulate (**Figure 7**). You can add more scenarios (a maximum of 5 per simulation) by pressing the + sign.

Please note that the changes of the scenario parameters will be affective from 2020 onwards.

The default setting is the current status of your screening programme according to the mandatory data you provided. You are able to change the following parameters:

**Sensitivity:** Select whether the test sensitivity should be improved. If you pick this option, the sensitivity will be increased by 5% across all age-groups.

**Target age**: Select the starting age and the maximum age at which women should be screened in this simulation.

The actual ages at which screening is performed in the simulation are determined by the starting age and the screening interval. Therefore, the last screening age of a woman can be slightly lower than the maximum screening age if the selected starting age and screening interval do not result in a screening invitation at this maximum age. For example, if the user selects a starting age of 50, a maximum age of 65 and an interval of 2 years, the last screening performed will be at age 50 + 7*2years = 64 years old.

**Screening interval:** Select how many years there should be between each screening round.

**Adherence:** Select how much the adherence should be increased. As a default, the screening coverage entered in the data template (sTable2) is used. Please note that, for each age-group, the current total examination coverage is calculated from column J in sTable2 (Examination coverage) PLUS the information on opportunistic screening participation from sTable11. If sTable11 is left empty, only the information from sTable2 is used.

By choosing an increase in adherence, the respective value (2.5%, 5%, 10%, 20%, 30% and 40%) will be added to the examination coverage of each of the age-groups equally. If, for example, the current coverage is 60% in the age-group 50-54, 65% in the age-group 55-59, 70% in the age-group 60-54, and 75% in the age-group 65-69 and you choose an increase in adherence of 10%, the coverage will increase to 70%, 75%, 80% and 85%, respectively.

**Stop screening:** If you would like to simulate the effect of a full stop of (organised) screening activities for your country or region from 2020 onwards, you can do so by choosing the “Stop screening” option under “adherence”. This option will overwrite all other parameter choices for this scenario.

No duplicate scenario can be selected.

Press “Start simulation” (**Figure 7**). Confirm that you are sure you want to start your simulation.

**
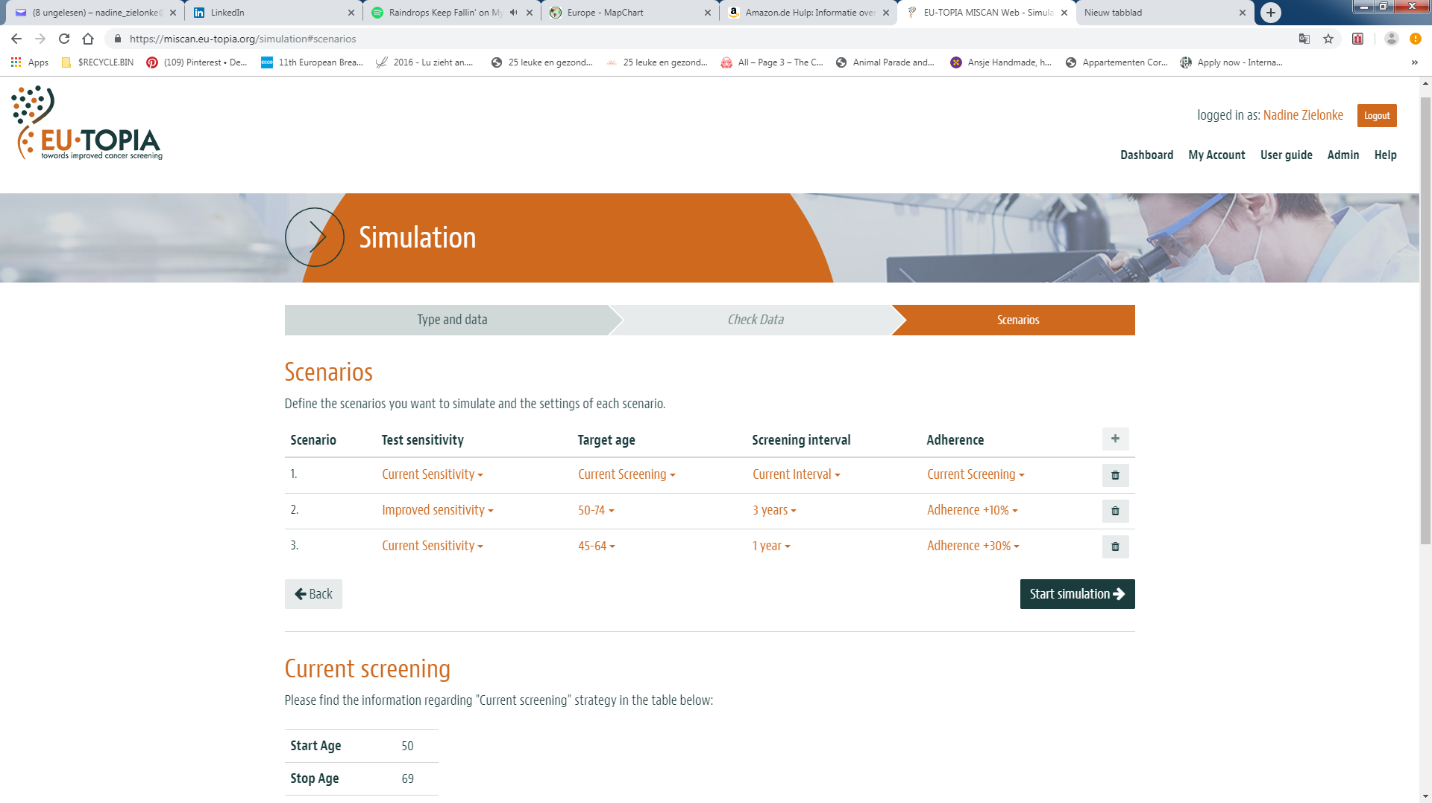
Figure 7**. EU-TOPIA evaluation tool, choose scenarios and start simulation.

Your simulation will be submitted for processing. You will receive an e-mail as soon as your simulation is finished.

**Results**

In this section it will be described how to download and interpret the results of the EU-TOPIA evaluation tool.

**Downloading the results**

Once your simulation is finished and the results are ready, you will receive an e-mail with a link. Following the link, you will reach your simulation online and be able to download a PDF report with the results **(Figure 8).**


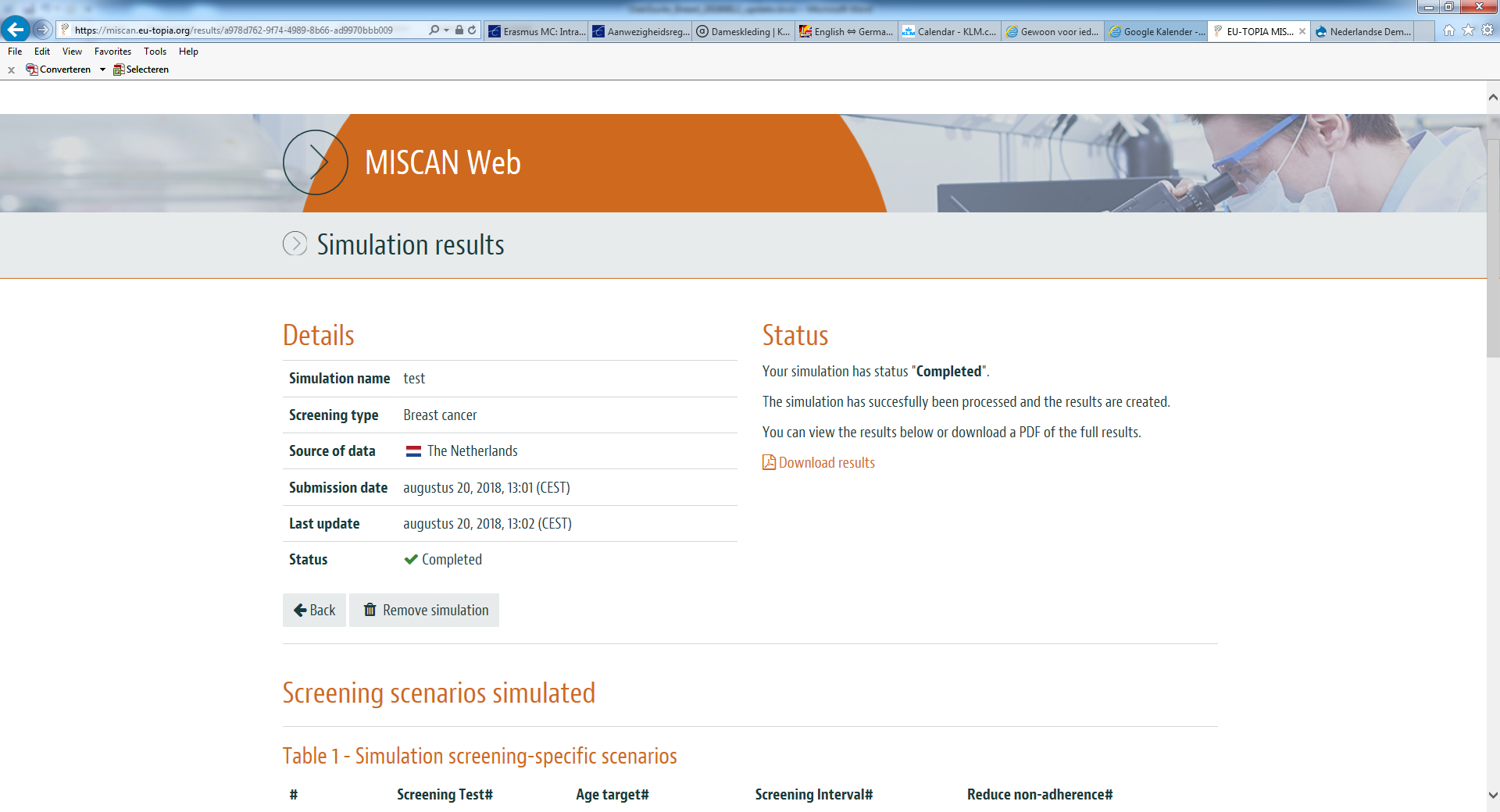
 **Figure 8**. EU-TOPIA evaluation tool, view and retrieve your simulation results

Alternatively, you can also find the results by going to the RESULTS section available in the EU-TOPIA evaluation tool dashboard (**Figure 9**). You will reach the results of the simulation by clicking on the specific simulation you are interested in.


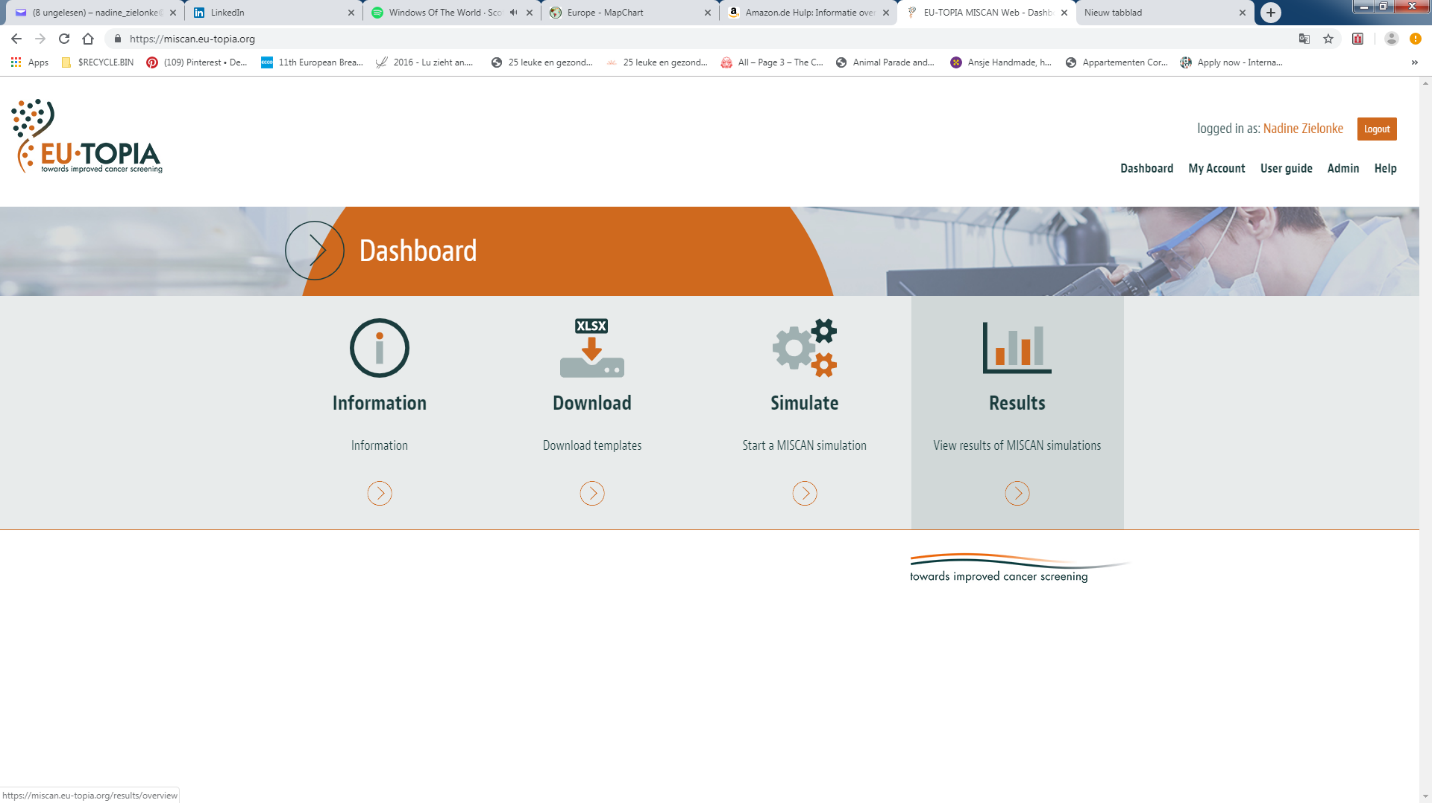


**Figure 9**. EU-TOPIA evaluation tool, access simulation results via dashboard

Here, an overview of all simulations you started is provided, including the status of the simulation, which can be:

**Submitted**: the server is currently running multiple other simulations, your simulation is in the queue.

**Processing**: your simulation is currently running on the server, your results will be ready soon.

**Failed**: something went wrong, the EU-TOPIA admins are informed and will contact you about a solution.

**Completed**: you will reach the results of the simulation by clicking on the simulation

On the page with the results of your simulation, you can find a summary of your simulated screening scenarios and first results: a graph with crude incidence rates per year (2020-2050), a graph with crude mortality rates per year (2020-2050), and a summary table with main outcomes for the population for the years 2020-2050, respectively.


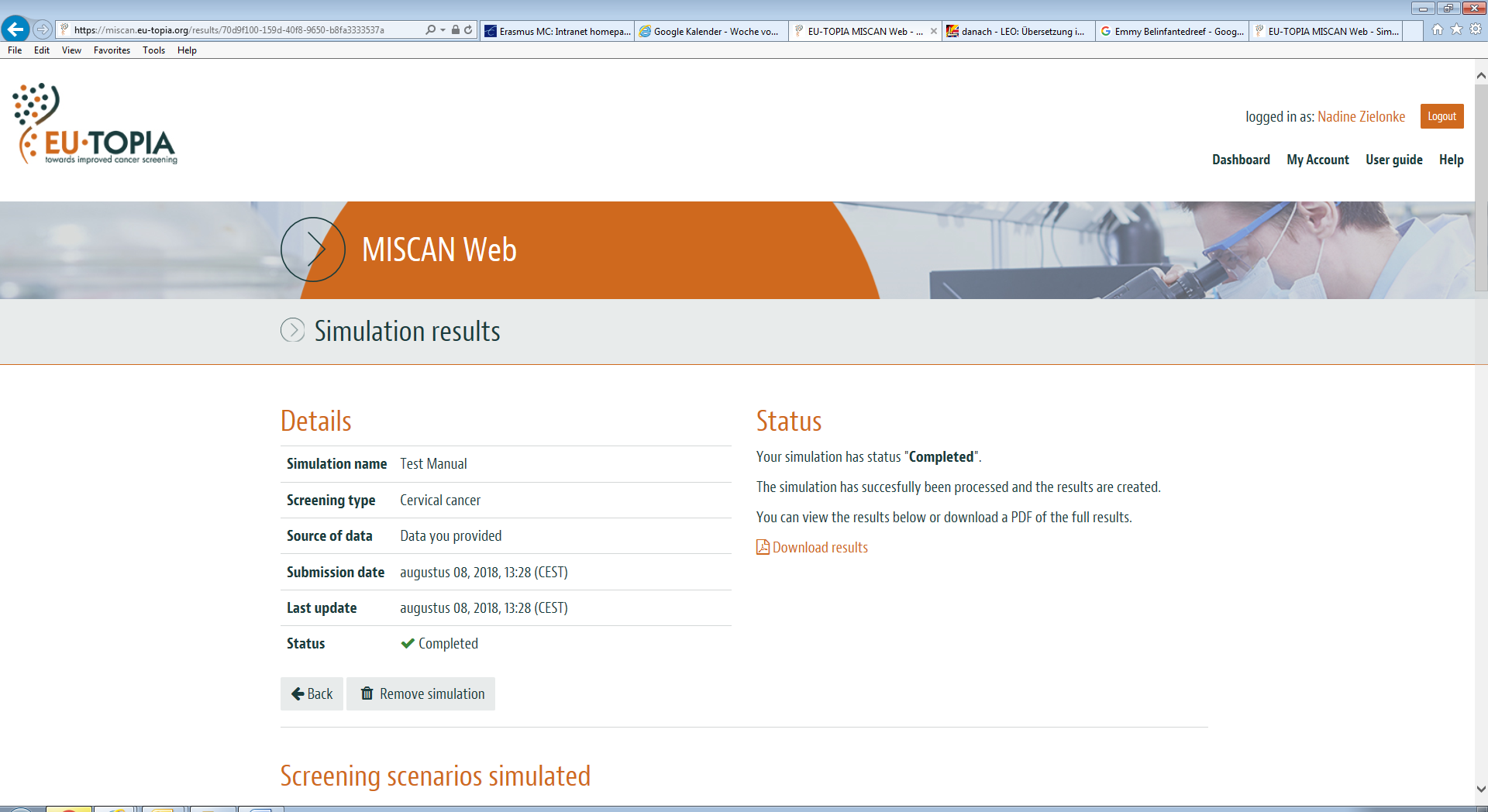


You can retrieve the PDF of the simulation report by pressing

If you want, you can start a new simulation.

**Description of the results.**

In the simulation, the life histories of 10,000,000 women were simulated, including Ductal Carcinoma In Situ (DCIS). The (new) screening strategy, including the adherence to screening, was applied. The EU-TOPIA evaluation tool produces several types of outputs, of which table 1 and 2 as well as figure 1 and 2 are displayed in your web browser, while the downloadable PDF presents the remaining more detailed tables as well.

**Box 1**

The first overview you see is a summary of data sources for the model inputs. This is based on the data you verified for your simulation – which are either based on the country-specific data you provided or on the data from the exemplary country of your region for non-mandatory data (see [Section 5.2.](#_Quality_check_of))

**Table1**

Here the settings of the selected scenario(s) are displayed. You can see the selected test sensitivity, target ages, screening interval and non-adherence reduction for each scenario. If current screening was selected, the settings as specified in the uploaded data template are used.

**Current screening strategy**

This table provides an overview of the current screening strategy and the adherence by age that all simulations are based on.

**Figure 1**

In this figure the crude breast cancer incidence rates per 100,000 women years between 2020 and 2050 are shown by age group for each of the scenarios.

**Figure 2**

In this figure the crude breast cancer mortality rates per 100,000 women years between 2020 and 2050 are shown by age group for each of the scenarios.

**Table2**

The results represents a summary of the screening outcomes in women aged 40 to 100 in 2020 to 2050 with the selected test sensitivity, target ages, screening interval and non-adherence reduction for each scenario.
The number of overdiagnosed breast cancers (DCIS and invasive) represent women that would not have been diagnosed during their lives if they had never been screened as a percentage of all diagnosed women. In addition, two ratios of harms and benefits are provided for each scenario: the number of screens needed to prevent one breast cancer death and the number of false positives per breast cancer death prevented.

**Tables 3-10**

In the downloadable PDF, all numbers from Table2 are available separated by age-group in tables 3-10.
